# mTOR regulates aerobic glycolysis through NEAT1 and nuclear paraspeckle-mediated mechanism in hepatocellular carcinoma

**DOI:** 10.7150/thno.72581

**Published:** 2022-04-24

**Authors:** Hong Zhang, Xiaoyang Su, Stephen K. Burley, X.F. Steven Zheng

**Affiliations:** 1 Rutgers Cancer Institute of New Jersey, Rutgers, The State University of New Jersey, 195 Little Albany Street, New Brunswick, NJ 08903, USA.; 2 Department of Pharmacology, Robert Wood Johnson Medical School, Rutgers, The State University of New Jersey, 675 Hoes Lane, Piscataway, NJ 08854, USA.; 3 Department of Medicine, Robert Wood Johnson Medical School, Rutgers, The State University of New Jersey, 125 Paterson Street, New Brunswick, NJ 08901.; 4 RCSB Protein Data Bank and Institute for Quantitative Biomedicine, Rutgers, The State University of New Jersey, Piscataway, 174 Frelinghuysen Road, NJ 08854 USA.; 5 Department of Chemistry and Chemical Biology, Rutgers, The State University of New Jersey, 174 Frelinghuysen Road, Piscataway, NJ 08854 USA.; 6 RCSB Protein Data Bank, Skaggs School of Pharmacy and Pharmaceutical Sciences and San Diego Supercomputing Center, University of California, San Diego, 9500 Gilman Drive, La Jolla, CA 92093 USA.

**Keywords:** mTOR, NEAT1, Paraspeckles, Splicing, Rapamycin, Normoxia, Hypoxia, Warburg Effect, HIF1, Aerobic Glycolysis, Hepatocellular carcinoma

## Abstract

**Background:** Hepatocellular Carcinoma (HCC) is a major form of liver cancer and a leading cause of cancer-related death worldwide. New insights into HCC pathobiology and mechanism of drug actions are urgently needed to improve patient outcomes. HCC undergoes metabolic reprogramming of glucose metabolism from respiration to aerobic glycolysis, a phenomenon known as the 'Warburg Effect' that supports rapid cancer cell growth, survival, and invasion. mTOR is known to promote Warburg Effect, but the underlying mechanism(s) remains poorly defined. The aim of this study is to understand the mechanism(s) and significance of mTOR regulation of aerobic glycolysis in HCC.

**Methods:** We profiled mTORC1-dependent long non-coding RNAs (lncRNAs) by RNA-seq of HCC cells treated with rapamycin. Chromatin immunoprecipitation (ChIP) and luciferase reporter assays were used to explore the transcriptional regulation of *NEAT1* by mTORC1. [U-^13^C]-glucose labeling and metabolomic analysis, extracellular acidification Rate (ECAR) by Seahorse XF Analyzer, and glucose uptake assay were used to investigate the role of mTOR-NEAT1-NONO signaling in the regulation of aerobic glycolysis. RNA immunoprecipitation (RIP) and NONO-binding motif scanning were performed to identify the regulatory mechanism of pre-mRNA splicing by mTOR-NEAT1. Myristoylated AKT1 (mAKT1)/NRAS^V12^-driven HCC model developed by hydrodynamic transfection (HDT) was employed to explore the significance of mTOR-NEAT1 signaling in HCC tumorigenesis and mTOR-targeted therapy.

**Results:** mTOR regulates lncRNA transcriptome in HCC and that NEAT1 is a major mTOR transcriptional target. Interestingly, although both NEAT1_1 and NEAT1_2 are down-regulated in HCC, only NEAT1_2 is significantly correlated with poor overall survival of HCC patients. NEAT1_2 is the organizer of nuclear paraspeckles that sequester the RNA-binding proteins NONO and SFPQ. We show that upon oncogenic activation, mTORC1 suppresses NEAT1_2 expression and paraspeckle biogenesis, liberating NONO/SFPQ, which in turn, binds to U5 within the spliceosome, stimulating mRNA splicing and expression of key glycolytic enzymes. This series of actions lead to enhanced glucose transport, aerobic glycolytic flux, lactate production, and HCC growth both *in vitro* and *in vivo*. Furthermore, the paraspeckle-mediated mechanism is important for the anticancer action of US FDA-approved drugs rapamycin/temsirolimus.

**Conclusions:** These findings reveal a molecular mechanism by which mTOR promotes the 'Warburg Effect', which is important for the metabolism and development of HCC, and anticancer response of mTOR-targeted therapy.

## Introduction

Hepatocellular carcinoma (HCC) is a major form of liver cancer, a leading cause of cancer death 1. Despite the reduced trend of overall cancer rate and cancer death, HCC has been steadily increasing in both incidence and as a cause of death during the last two decades 2. Specifically, the death rate for liver cancer nearly doubled in the US over this period 3. Standard care for early HCC patients includes surgery, chemotherapy, and liver transplantation with surgery as the primary curative option 4. However, HCC is commonly diagnosed at advanced stages that are not amenable for surgery. Multikinase inhibitors (e.g., sorafenib, regorafenib) and immune checkpoint blockade antibodies are used for treatment of advanced HCC. Regrettably, these therapies suffer from limited durable response rate and survival benefit 5, 6. New systemic drugs and therapeutic strategies are urgently needed to improve clinical outcomes in HCC.

Mechanistic target of rapamycin (mTOR) is a master controller of cell growth and metabolism 7, 8. mTOR complex 1 (mTORC1) is the molecular target of rapamycin and rapamycin analogs (*e.g*., everolimus, temsirolimus). Everolimus and temsirolimus are US FDA-approved drugs for breast, renal and neuroendocrine cancers. Oncogenic activation of mTOR signaling is commonly observed in clinical HCC samples 9, 10. Aberrant activation of mTORC1 pathway is sufficient to drive hepatocarcinogenesis in genetically engineered and hydrodynamic transfection mouse models 11-14. However, recent clinical trials did not achieve desirable endpoints for rapamycin analogs everolimus and temsirolimus in advanced HCC patients 15, 16. Therefore, identifying new treatment strategies such as combinational therapy is necessary for improving the efficacy of rapamycin analogs in order to realize the benefits of mTOR-targeted therapies into the clinic.

mTORC1 pathway promotes cancer growth in part by reprogramming glucose metabolism from respiration (oxidative phosphorylation) to aerobic glycolysis 17, 18, a phenomenon known as 'Warburg Effect', which is a hallmark of cancer that supports rapid cancer cell growth, survival, and invasion 19. It has been reported that mTOR stimulates glycolysis through HIF1α-dependent transcription of glycolytic enzymes 20, 21. However, HIF1α is a hypoxia-induced transcription factor that is rapidly degraded through ubiquitin-dependent degradation under normoxic conditions 22, 23. Activation of HIF1α expression by mTORC1 occurs under hypoxic conditions, such as ischemic tumor microenvironment 24, 25. Thus far, the precise mechanism(s) by which mTOR promotes aerobic glycolysis remains unknown.

Previous studies showed that mTORC1 regulates expression of mRNA, rRNA, and tRNA genes involved in cellular growth 26. In this study, we show that mTORC1 regulates the long non-coding RNA (lncRNA) transcriptome. One of mTORC1 target lncRNAs is NEAT1, an organizer of nuclear paraspeckles that are formed by polymerization of NEAT1 with RNA binding proteins NONO and SFPQ 27. However, the regulation and biological functions of nuclear paraspeckles remain poorly understood. Interestingly, expression of NEAT1, particularly the NEAT1_2 isoform is significantly down-regulated in human HCC tumors, which is correlated with poor overall survival of HCC patients. Herein, we show that mTORC1 suppresses NEAT1_2 expression and NEAT1_2-dependent nuclear paraspeckle biogenesis, which promotes mRNA splicing and expression of key glycolytic enzymes and the Warburg Effect in HCC. Importantly, this mTORC1 signaling mechanism contributes to aberrant liver cancer metabolism, liver tumor development, and response to mTORC1-targeted therapy.

## Materials and methods

### Cell culture, plasmid construction and transfection

Human HCC cell lines SNU423 and SNU387 were cultured in RPMI 1640 Medium (Thermo Fisher Scientific, USA) plus 10% fetal bovine serum (FBS) (Biological Industries, Israel). TSC1^+/-^ MEF, TSC1^-/-^ MEF cells and human HCC cell lines Huh1, Hep3B, MHCC-97H, C3A, Huh7 and PLC/PRF/5 were maintained in high-glucose DMEM (Thermo Fisher Scientific, USA) plus 10% FBS. Cells were incubated at 37 °C in a humidified chamber containing 5% CO2. None of the cell lines are categorized as misidentified cell lines by ICLAC and NCBI Biosample.

Plasmids used in this project, including pT3‐EF1α, pT3‐EF1α‐HA-myr-AKT1, pT3‐EF1α‐NRAS^V12^ and pCMV/sleeping beauty transposase were generous gifts of Dr. Xin Chen, University of California, San Francisco. shRNA against NEAT1_2 (pT3-EF1α‐shNEAT1: 5'-GGGTAAATCTCAATCTTAA-3') was cloned into pT3-EF1α plasmid *via* the Gateway PCR cloning strategy (Thermo Fisher Scientific, USA). GFP-tagged NONO plasmid was purchased from Sino Biological (USA). The pTN24 splicing reporter plasmid was a gift from Ian C. Eperon (University of Leicester, Leicester, UK). All plasmids were purified using the Endotoxin free Maxi prep kit (Sigma-Aldrich, USA). Validated siRNAs 28 for Human cell lines were purchased from Sigma (USA). siRNA were transfected into HCC cells using Lipofectamine 3000 (Invitrogen, USA) as described previously 29, 30.

To generate double-allele ΔRRE SNU423 cell lines, 2 sgRNAs (one targeting upstream and one targeting downstream of the RRE region)(Genescript) were transfected into SNU423 cells, which was followed by single clonal selection. Cell clones were lysed by QuickExtract™ DNA Extraction Solution (Biosearch Technologies) by incubating at 55 °C for 20 min and then 95 °C for 2 min. Genomic DNA was analyzed by PCR using primers flanking guide RNA target sites (fwd: 5'- TCAAAAGCGAATGGATCCCA-3' and Rev: 5'- CGGTTCAAGTAACCAGAATG-3'). The positive clones were confirmed by Sanger sequencing.

### Mice and hydrodynamic tail vein injection

All animal protocols were performed with approval of the Institutional Animal Care and Use Committee (IACUC) of Rutgers, the State University of New Jersey. FVB/NJ mice were purchased from Jackson Laboratory (Bar Harbor, ME) and used for hydrodynamic tail vein injection as previously described 31, 32. pT3-EF1α-HA-myr-AKT (4µg) + pT3-EF1α-NRAS^V12^ (4 µg) alone or with pT3-EF1α‐shNEAT1 was hydrodynamically injected into FVB/NJ mice. Plasmid DNA was diluted in 2 ml saline (0.9% NaCl) together with sleeping beauty transposase (SB) in a ratio of 25:1 and injected into the lateral tail vein of the mice in 5 to 7 seconds. PBS injection was used as controls. Temsirolimus (LC laboratories, USA) (6mg/kg/day) or vehicle was intraperitoneally administered for 7 weeks after hydrodynamic injection of 26 days. Mice were monitored for liver tumor development as palpable abdominal masses and euthanized as described in the Results section.

### Histology and immunohistochemistry (IHC)

Liver specimens were fixed in 10% paraformaldehyde and then embedded in paraffin. Immunohistochemistry was performed as previously described 33-35. Tissue slides were deparaffinized in xylenes and rehydrated in graded ethanol and then boiled in 10 mM citrate buffer (pH 6.0) for 30 min by placement in a microwave, followed by cool down at room temperature. 3% hydrogen peroxide in methanol was used to quench endogenous peroxidase activity by incubation for 10 min. After blocking with the blocking solution (PBST/5% normal goat serum), slides were then incubated with primary antibodies overnight at 4 °C. Subsequently, tissue slides were incubated with SignalStain® Boost IHC Detection Reagent (Cell Signaling Technology, USA) for 30 min at RT. Finally, tissue sections were developed with SignalStain® DAB Substrate (Cell Signaling Technology, USA) and counterstained with hematoxylin.

### Statistical analysis

Statistical data analyses were performed by SPSS 11.0 software (SPSS Inc., USA) and Graph Pad Prism 8.0 for windows (Graph Pad Prism, Inc., San Diego, CA, USA). Experimental data were expressed as mean ± SD or SEM. One-way ANOVA test and Student's t-test were conducted to evaluate the variables of different groups. A nonparametric Spearman correlation test was used to compare the correlation between NEAT1 expression and the activity of mTOR pathway. Repeated measures ANOVA was performed to analyze proliferation of cultured cells. Kaplan-Meier plots and log-rank test were performed to compare cancer specific survival rates between patients with high and low NEAT expression. p < 0.05 was considered as statistically significant. Statistical details and methods used are indicated in the figure legends, text or methods.

See Supplementary Materials for Additional Materials and Method.

## Results

### mTORC1 regulates lncRNA transcriptome in liver cancer cells

**Figure 1:**
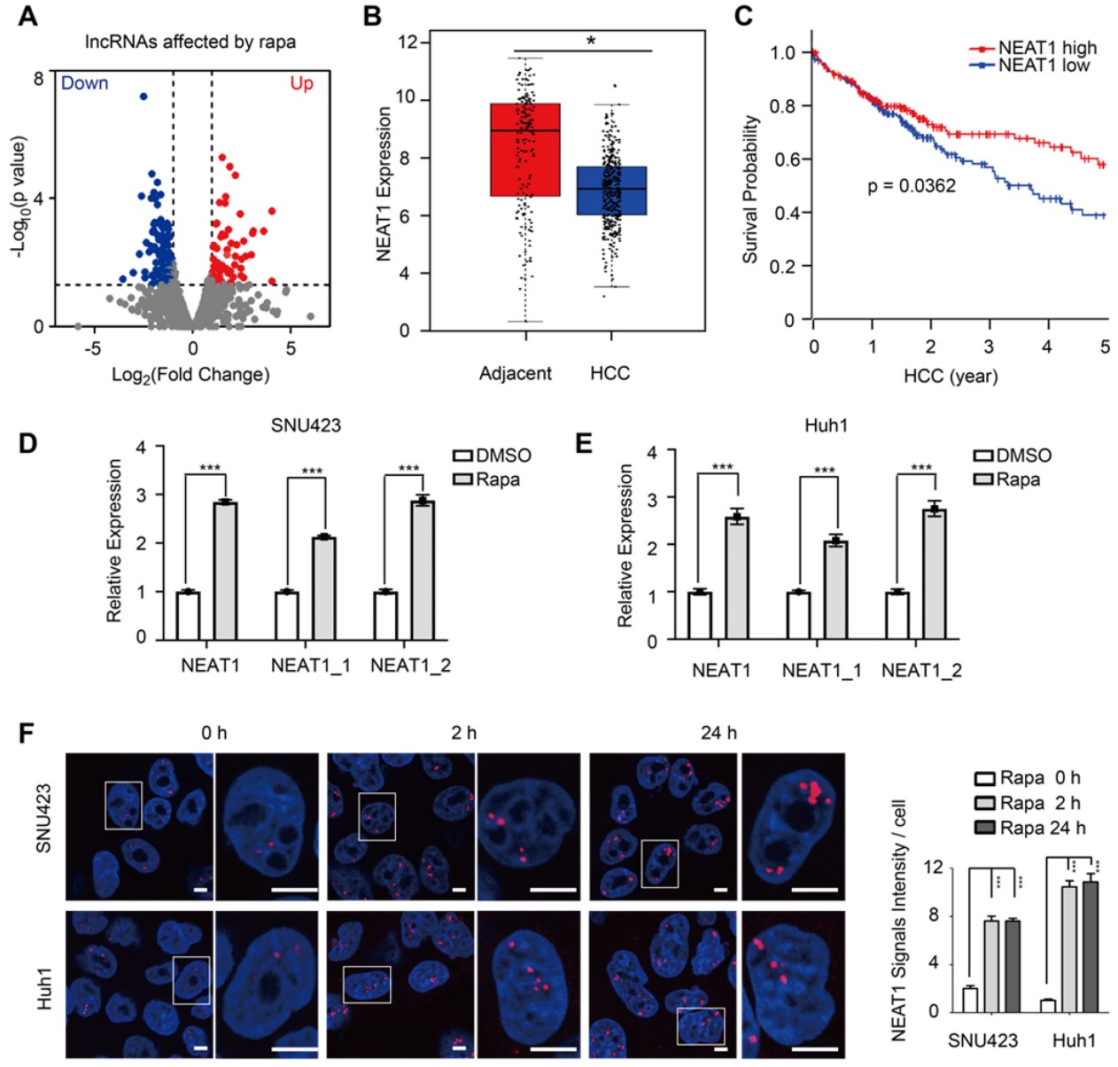
** mTORC1 regulates lncRNA transcriptome, NEAT1 expression and nuclear paraspeckle biogenesis. (A)** Identification of mTORC1-regulated lncRNAs by RNA-seq of SNU423 cells treated with 100 nM rapamycin for 2 h. Shown is volcano plot of differentially-expressed lncRNAs (FC ≥2, *p* ≤0.05). **(B)** Down-regulation of NEAT1 mRNA in human HCC tumors. NEAT1 RNA expression was analyzed in 369 primary HCC and 160 normal liver tissues (GEPIA dataset). *p* value was determined by unpaired two-tailed Student's *t* test (bar represents mean value). * *p* < 0.05. **(C)** HCC patients with low NEAT1 expression have poor overall survival. Kaplan-Meier analysis was used to compare overall survival of HCC patients with high and low NEAT1 expression. *p* value was calculated using the two-sided log-rank test. **(D and E)** Rapamycin rapidly induces expression of total NEAT1, NEAT1_1 and NEAT1_2 in HCC cells. SNU423 (D) and Huh1 (E) cells were treated with 100 nM rapamycin for 2 h and RNA expression was analyzed by qRT-PCR. Values are normalized against ACTIN mRNA. Bar graphs represent mean ± SEM (n = 3). P value was determined by two-tailed unpaired t test. *** *p* < 0.001. **(F)** Rapamycin promotes paraspeckle biogenesis in HCC cells. SNU423 cells were treated with 100 nM rapamycin for different times. Paraspeckles were stained by RNA-FISH with NEAT1_2 probe (red) and nuclei were counterstained with DAPI (blue). Right panel shows quantification of the result (paraspeckles/nucleus view). Bar graphs represent mean ± SEM (n = 5) and p value was determined by one-way ANOVA followed by Tukey's multiple comparisons test. *** *p* < 0.001.

Because mTORC1 broadly controls nuclear gene expression 36, we asked if mTORC1 regulates lncRNAs by performing RNA-seq of SNU423 cells in response to mTORC1 inhibition by rapamycin. Rapamycin treatment significantly altered the expression of 202 lncRNAs (≥2-fold), of which 75 and 127 were up-regulated and down-regulated, respectively (Figure 1A, Table S1), indicating that mTORC1 broadly regulates lncRNA transcriptome. We next performed a secondary screening of the mTORC1-dependent lncRNAs for those with aberrant expression in liver cancer by analyzing a TCGA transcriptome dataset derived from 369 primary HCC tumors and 160 normal livers 37. Among mTORC1 target lncRNAs, NEAT1 was notably down-regulated in the setting HCC *versus* normal liver (Figure 1B). Moreover, low NEAT1 expression was significantly correlated with poor overall survival (OS) of HCC patients (p = 0.0362) (Figure 1C), suggesting that NEAT1 plays an important role in HCC. Thereafter, we focused on understanding regulation of NEAT1 by mTORC1 and the significance of this regulation. There are two completely overlapping NEAT1 isoforms encoded by the same *NEAT1* gene: NEAT1_1 and NEAT1_2 (Figure S1A). We analzyed differential expression of the two isoforms in the same cohort of HCC tumors and normal livers 38. NEAT1_2 was down-regulated in HCC and low NEAT1_2 expression was significantly correlated with poor OS of HCC patients (p < 0.001) (Figure S1B). Although NEAT1_1 was also moderately down-regulated in HCC, its expression was not significantly associated with poor OS of HCC patients (Figure S1C). These observations indicate that NEAT1_2 rather than NEAT1_1 plays an important role during liver cancer progression.

### mTOR suppresses NEAT1 expression and paraspeckle biogenesis

Consistent with the RNA-seq results, acute inhibition of mTORC1 signaling by rapamycin rapidly up-regulated expression of both NEAT1_1 and NEAT1_2 in Huh1 and SNU423 cells, albeit rapamycin induction of NEAT1_2 at moderately higher level than NEAT1_1 (Figure 1D-E, Figure S2A). Long-term rapamycin treatment sustained high NEAT1 expression (Figure S2B-C). NEAT1_2 is the organizer and backbone of paraspeckles 27. We therefore investigated possible regulation of paraspeckle biogenesis by mTORC1. HCC cells treated with rapamycin and paraspeckles were visualized by RNA-fluorescence *in situ* hybridization (RNA-FISH) using a NEAT1_2 specific probe. Rapamycin treatment were found to rapidly increase paraspeckles (Figure 1F). These results demonstrate that mTORC1 negatively regulates NEAT1_2 expression and paraspeckle biogenesis.

### mTORC1 negatively regulates NEAT1 expression *in vitro*

**Figure 2:**
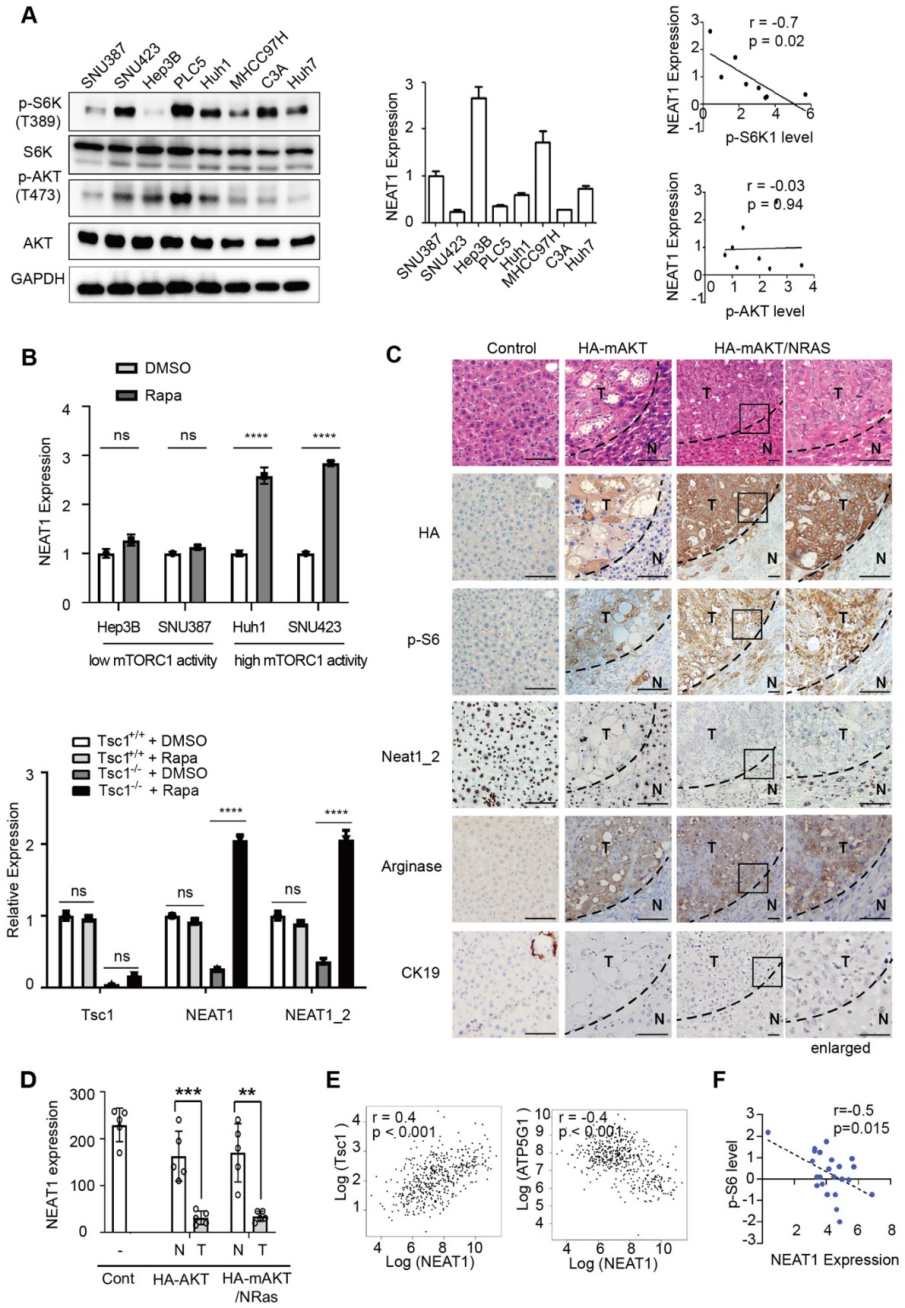
** Hyperactivated mTORC1 suppresses NEAT1 expression *in vitro* and *in vivo*. (A)** NEAT1 expression is negatively correlated with mTORC1 signaling, not mTORC2 signaling in HCC cell lines. mTORC1 and mTORC2 signaling were measured by immunoblot of p-S6K(T389) and p-Akt (S473), respectively (left panel) in a panel of HCC cell lines (SNU387, SNU423, Hep3B, PLC5, Huh1, MHCC97H, C3A, Huh7). NEAT1 expression was determined by qRT-PCR (middle panel) in the same panel of HCC cell lines. Correlation analysis of NEAT1 expression with mTORC1 and mTORC2 signaling was determined by nonparametric Spearman correlation test (right panels). **(B)** Hyperactive mTORC1 represses NEAT1 expression. HCC cells with high mTORC1 activity (Hep3B, SNU387) and low mTORC1 activity (Huh1, SNU423) were treated without or with 100 nM rapamycin for 2 h (upper panel), and analyzed for NEAT1 expression by qRT-PCR. *WT* and *Tsc1^-/-^* MEF cells were treated without or with 100 nM rapamycin for 2 h and analyzed for NEAT1 expression by qRT-PCR (lower panel). NEAT1 expression was calculated as relative to ACTIN. Bar graphs represent mean ± SEM (n = 3). Data are shown as mean ± SEM (n = 3); Statistical significance was tested using two-tailed unpaired t test. *** *p* < 0.001, **** *p* < 0.0001, ns, not significant. **(C)** Oncogenic activation of mTORC1 signaling represses NEAT1 expression *in vivo*. Mouse livers were transfected with a vector control, HA-mAKT or HA-mAKT plus NRAS through hydrodynamic transfection (HDT) in mice. Liver tissues were analyzed by HE staining, anti-HA IHC staining, and Neat1_2 RNAscope staining. Mouse liver tumor tissues were stained by IHC positively for the HCC marker Arginase, but negatively for the cholangiocarcinoma marker CK19. **(D)** Quantification of Neat1_2 staining results in (C). Data are shown as mean ± SEM (n = 5); Statistical significance was tested using two-tailed unpaired *t* test. *** *p* < 0.001, ** *p* < 0.01. N, normal liver; T, tumor. **(E)** NEAT1 expression is negatively correlated with mTORC1 signaling in human HCC tumors. Correlation analysis of NEAT1 RNA expression with TSC1 mRNA (left panel) and ATP5G1 mRNA (right panel) in 369 primary human HCC tumors (http://gepia.cancer-pku.cn/). *p* value was analyzed by nonparametric Spearman correlation test. **(F)** NEAT1 expression is negatively correlated with mTORC1 signaling in HCC cells as determined protein expression analysis. NEAT1 expression and p-S6 (S235/236) was performed in a CCLE HCC panel. Protein and NEAT1 expression data was downloaded from the CCLE portal (https://portals.broadinstitute.org/ccle). The correlation score was analyzed using Pearson correlation test.

mTORC1 pathway is excessively activated in human HCC, driving liver tumorigenesis 9, 10. To explore the relationship between mTOR signaling and NEAT1 expression in HCC, we examined the level of mTORC1 (p-S6K) and mTORC2 (p-AKT) signaling (left panel, Figure 2A), and NEAT1 expression (middle panel, Figure 2A) in a panel of HCC cell lines. NEAT1 expression showed a strong inverse correlation with mTORC1 signaling (r = -0.7, p = 0.02), but no apparent correlation with mTORC2 signaling (r = -0.03, p = 0.94) (right panel, Figure 2A). Consistently, rapamycin preferentially stimulated NEAT1 expression in HCC cells (Huh1 and SNU423) with elevated mTORC1 signaling (Figure 2A, upper panel of Figure 2B, Figure S3A). Tsc1 knockout (*Tsc1^-/-^*) in MEF cells resulted in mTORC1 hyperactivation (Figure S3B) 39 and down-regulation of Neat1 and Neat1_2 expression (lower panel of Figure 2B, Figure S3B). Moreover, rapamycin treatment robustly induced Neat1 and Neat1_2 (lower panel of Figure 2B, Figure S3B). Consistently, TSC1 knockdown in HCC cells increased mTORC1 activity and further decreased NEAT1 expression (Figure S3C-D). These results show that hyperactive mTORC1 signaling represses NEAT1_2 expression.

### mTORC1 negatively regulates NEAT1 expression *in vivo* and in clinical samples

To explore the relationship of mTORC1 signaling and NEAT1 expression *in vivo*, we analyzed mouse liver tumors driven by constitutively active, myristoylated AKT (mAKT) or mAKT in combination with NRAS^V12^ (mAKT/NRAS) in a hydrodynamic transfection (HDT) model 11. In this model, mAKT and mAKT/NRAS are specifically and stably expressed in hepatocytes, activating mTORC1 and driving HCC development, as indicated by elevated p-S6 and HCC marker arginase and cholangiocarcinoma marker CK19, respectively (Figure 2C) 11. Neat1_2 expression was markedly down-regulated in liver tumors driven by hyperactive mTORC1 signaling, compared with control normal liver or tumor adjacent liver tissues (Figure 2C-D). Hence, hyperactive mTORC1 signaling suppresses Neat1_2 expression and paraspeckles *in vivo*. To ask whether or not mTORC1 signaling regulates NEAT1 in a clinical setting, we analyzed a GEPIA transcriptome dataset derived from 369 human primary HCC tumors 40. The expression of TSC1, an upstream negative regulator of mTORC1, was found to be positively correlated with NEAT1 levels (left panel, Figure 2E). In contrast, ATG5G1, a marker for mTORC1 activation 41, was negatively correlated with NEAT1 levels (right panel, Figure 2E). Consistently, the expression of TSC1 showed positive correlation with the level of NEAT1_2, and to a lesser degree, with that of NEA1_1 in primary HCC tumors (Figure S3E). Moreover, integrated phosphoproteomic and transcriptomic analysis of Cancer Cell Line Encyclopedia (CCLE) datasets showed that p-S6 was negatively correlated with NEAT1 expression (Figure 2F). Together, these results provide *in vivo* and clinical evidence that hyperactive mTORC1 signaling negatively regulates NEAT1 expression.

### mTORC1 acts at *NEAT1* promoter and negatively regulates *NEAT1* transcription

**Figure 3:**
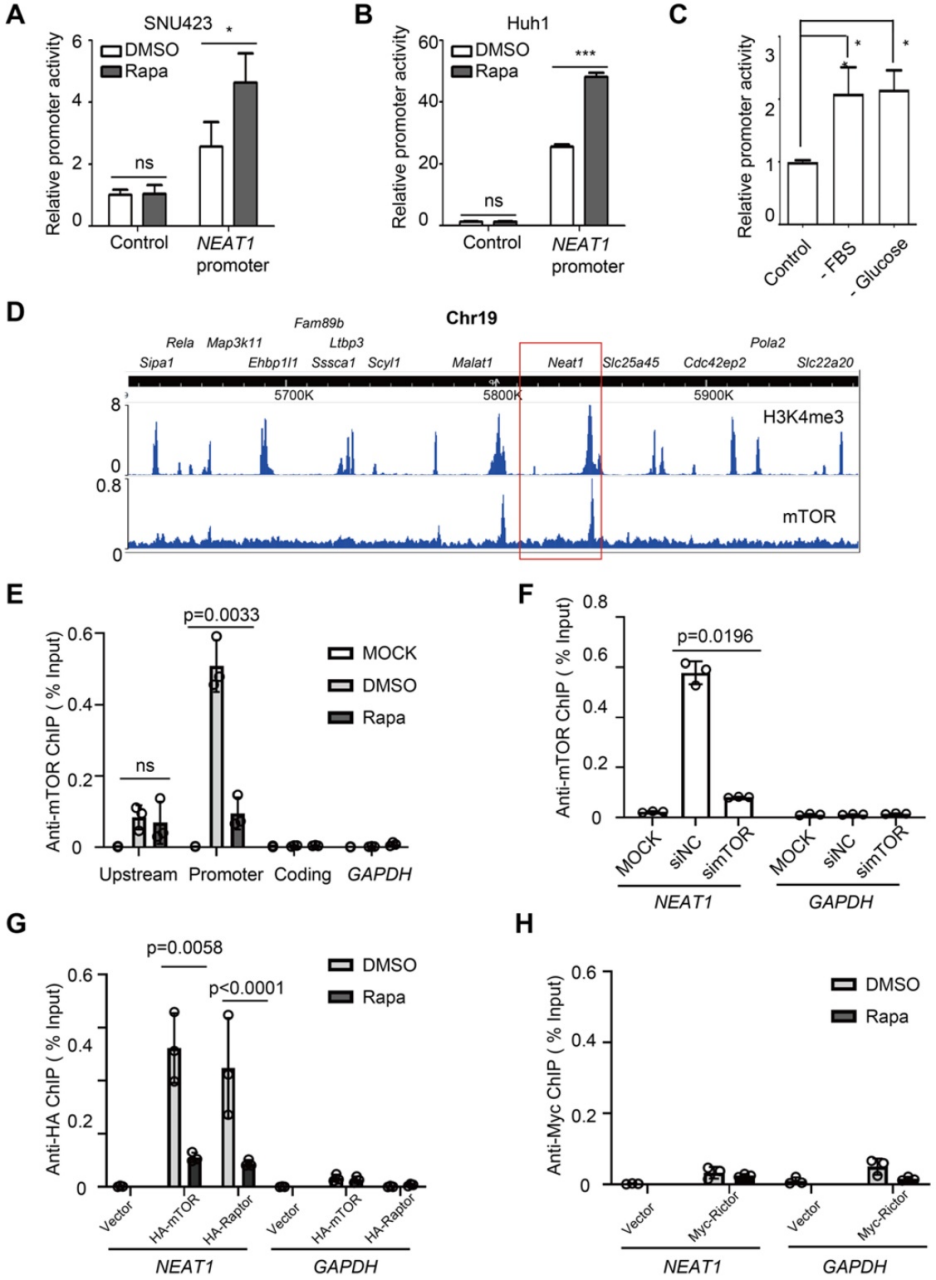
** mTORC1 acts at NEAT1 promoter and negatively regulates NEAT1 transcription. (A-B)** Rapamycin stimulates *NEAT1* promoter activity. Activity of *NEAT1* promoter-driven luciferase reporter was measured in SNU423 (A) and Huh1 (B) cells treated with or without 100 nM rapamycin for 24 h. Mean ± SEM (n = 3), Unpaired two-tail *t* test; **(C)** *NEAT1* promoter activity is regulated by glucose and growth factors. SNU423 cells transfected with *NEAT1* promoter luciferase reporter were starved from fetal bovine serum (FBS) or glucose. Data (mean ± SEM, n = 3) were analyzed by unpaired two-tail *t* test. **(D)** Shown are peaks of mTOR-binding and histone H3K4me3 across a region of Chromosome 19 in mouse liver as determined by analysis of the ChIP-seq (mTOR dataset: GSM1067407; H3K4me3 dataset: GSM1970920). Boxed region shows *Neat1* locus. **(E)** mTOR binds to the *NEAT1* promoter in a rapamycin-sensitive manner in HCC cells. SNU423 cells were treated without or with 100 nM rapamycin for 2 h. Anti-mTOR ChIP was performed and the results were analyzed by qRT-PCR. Blue boxes indicate *NEAT1* coding, promoter and upstream region used for ChIP analysis. *GAPDH* promoter was used as a negative control. % Input = 100*2^(Adjusted input - Ct (IP). Mean ± SEM (n = 3), unpaired two-tail *t* test. **(F)** mTOR binding to *NEAT1* promoter is blocked by mTOR knockdown in HCC cells. SNU423 cells were transfected with mTOR siRNA (simTOR) or control siRNA (siNC). mTOR binding to *NEAT1* promoter was assayed by anti-mTOR ChIP. **(G)** mTORC1 binds to *NEAT1* promoter in a rapamycin-sensitive manner. HA-mTOR or HA-Raptor was transiently expressed in SNU423 cells and then treated without or with rapamycin. HA-mTOR and HA-Raptor binding to *NEAT1* promoter was assayed by anti-HA ChIP. **(H)** mTORC2 does not bind to *NEAT1* promoter in HCC cells. Myc-Rictor was transiently expressed in SNU423 cells and then treated without or with rapamycin. Myc-Rictor binding to *NEAT1* promoter was assayed by anti-Myc ChIP. Mean ± SEM (n = 3), unpaired two-tail *t* test. *** *p* < 0.001. ns, not significant.

To ask whether mTORC1 regulates NEAT1 promoter activity, we constructed a *NEAT1* promoter-driven luciferase reporter and assayed its activity in HCC cells. The luciferase activity increased in response to rapamycin treatment (Figure 3A-B). Starvation from serum and glucose also activated the NEAT1 promoter-driven luciferase activity (Figure 3C). These results indicate that mTORC1 represses *NEAT1* transcription in response to nutrient signals. Deletion mapping analyses revealed a rapamycin response element (RRE) in *NEAT1* promoter required for rapamycin-stimulation (Figure S4A). To demonstrate the physiological relevance of the reporter assay, we used CRISPR/cas9 to disrupt the RRE from the native promoter region of *NEAT1* genomic locus in SNU423 cells. Indeed, disruption of RRE was sufficient to blunt rapamycin-stimulated NEAT1_2 expression in HCC cells (Figure S4B-C). mTORC1 is known to act at the promoter of rRNA, tRNA and certain mRNA genes 42-44. We hence analyzed an anti-mTOR chromatin immunoprecipitation-sequencing (ChIP-seq) dataset derived from mouse livers 45. The result revealed a major mTOR-binding peak in the promoter region of *Neat1* that overlapped with an H3K4me3 peak (Figure 3D), a transcriptionally active chromatin marker, indicating that mTOR binds to a main regulatory region for *Neat1* promoter. Another mTOR binding peak was found at the promoter of *Malat1*, also a lncRNA target of mTORC1 identified in our screen (Figure 3D). In contrast, no significant mTOR peak was seen with other neighboring genes on Chromosome 19 (Figure 3D). Anti-mTOR ChIP assay detected strong mTOR binding to the promoter region of *NEAT1* promoter in a rapamycin-sensitive manner in human liver cancer cells (Figure 3D-E). Little or no mTOR binding was detected at the 5'-upstream region and coding region of *NEAT1,* or the promoter region of *GAPDH* (Figure 3E). The anti-mTOR ChIP signal was also diminished after mTOR knockdown (Figure 3F, Figure S5A). These results demonstrate the specificity of the ChIP assays. Significant anti-HA ChIP signals were detected at *NEAT1* promoter (not *GAPDH* promoter) in a rapamycin-sensitive manner in human HCC cells expressing HA-mTOR and HA-Raptor (Figure 3G, Figure S5B-C). In contrast, no significant binding of Myc-Rictor was detected at *NEAT1* promoter (Figure 3H, Figure S5D). Collectively, our study demonstrates that mTORC1, not mTORC2, represses *NEAT1* promoter in a rapamycin-sensitive manner.

### NEAT1_2 mediates mTORC1 regulation of NONO-U5 SNRNP interaction

**Figure 4:**
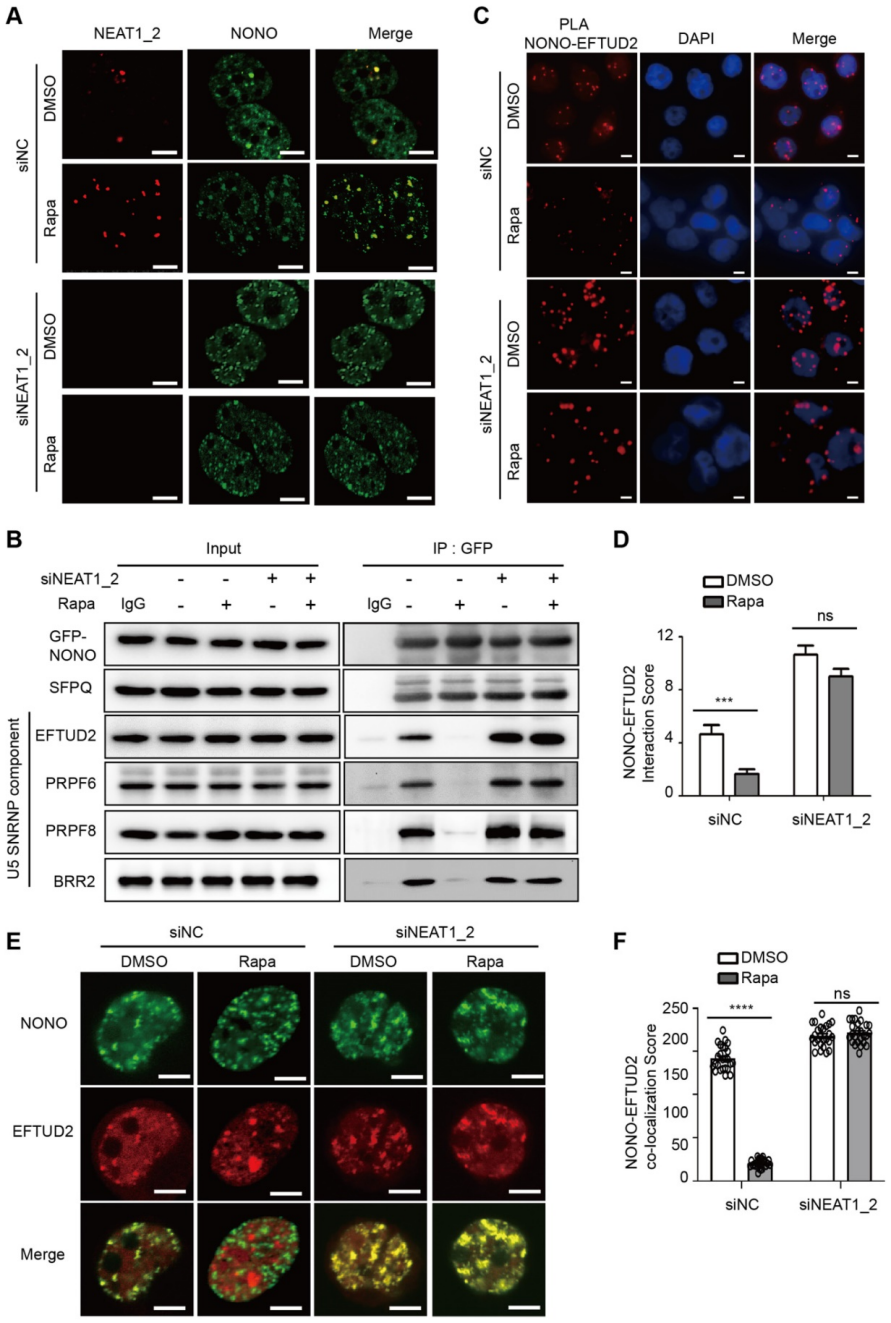
** mTORC1 regulates NONO-U5 SNRNP interaction in a paraspeckle-dependent manner. (A)** Rapamycin promotes NEAT1-dependent adsorption of NONO to paraspeckles. SNU423 cells transfected with NEAT1_2 or control siRNA were treated without or with 100 nM rapamycin for 24 h. Localization of NEAT1 and NONO were analyzed by FISH and IF staining, respectively. The nuclei were counterstained by DAPI. Confocal images are shown: Scale bar, 10 µm. **(B)** Rapamycin disrupts NONO interaction with U5 spliceosome in a NEAT1-dependent manner. GFP-NONO was assayed for interaction with U5 components by co-IP from extracts of SNU423 cells treated without or with 100 nM rapamycin. **(C and D)** Rapamycin disrupts NONO interaction with EFTUD2 in intact SNU423 cells, as measured by proximity ligation assay (PLA). PLA was performed in SNU423 cells treated without or with 100 nM rapamycin in the absence or presence of NEAT1_2 knockdown. Scale bar, 10 µm (C). Quantification of NONO-EFTUD2 interaction by PLA (D). Bar graphs represent mean ± SEM (n = 5) and p value was determined by unpaired two-tail *t* test. *** p < 0.001. ns, not significant. **(E and F)** Rapamycin disrupts NONO-EFTUD2 co-localization in a NEAT1-dependent manner. SNU423 cells were treated with or without 100 nM rapamycin in the absence or presence of NEAT1_2 knockdown. NONO and EFTUD2 localization was analyzed by IF (E) and the results were quantified for co-localization (F). Scar bar, 10 µm. Bar graphs represent mean ± SEM (n = 25, number of cells counted) and p value was determined by unpaired two-tail *t* test. **** p < 0.0001. ns, not significant.

During paraspeckle biogenesis, NEAT1_2 RNA polymerizes NONO and SFPQ, and quantitatively packages them into paraspeckles 27. Consistent with negative regulation of paraspeckle biogenesis by mTORC1, paraspeckle levels were normally very low in HCC cells and NONO was largely dispersed in the nucleoplasm (Figure 4A). Rapamycin treatment induced expression of NEAT1 and recruitment of NONO into paraspeckles (Figure 4A). The rapamycin effect was abrogated by NEAT1_2 knockdown (Figure 4A). Pre-mRNA splicing is catalyzed in a stepwise fashion by the U1-U5 spliceosomes. NONO was previously shown to bind to U5 small nuclear RNA (snRNA) in an *in vitro* reconstituted assay, triggering formation of stable splicing subcomplexes by recruiting core splicing components to pre-mRNA 46. NONO was normally found to be associated with U5 spliceosome as judged by co-immunoprecipitation of NONO with EFTUD2, PRPF6, PRPF8 and BRR2, components of U5 spliceosome (Figure 4B). NONO association with U5 spliceosome was disrupted by rapamycin (Figure 4B). However, NONO remained bound to U5 spliceosome in the presence of rapamycin after NEAT1_2 knockdown (Figure 4B). Rapamycin does not affect NONO protein or mRNA expression (Figure S6A-B) or formation of stable NONO-SFPQ complex (Figure 4B, Figure S6C). Proximity ligation assays (PLA), a method to detect protein-protein interactions in intact cells, also showed that NONO interacted with EFTUD2, which was disrupted by rapamycin in a NEAT1-dependent manner (Figure 4C-D). Consistently, NONO co-localized with EFTUD2 in the nucleus in a rapamycin-sensitive and NEAT1_2-dependent manner (Figure 4E-F). Interestingly, NEAT1_2 knockdown not only abrogated the rapamycin effect, but also further enhanced NONO interaction with EFTUD2 (Figure 4C-E), an effect likely due to the elimination of the basal level of paraspeckles. Collectively, these results show that mTORC1 regulates binding of NONO to the U5 spliceosome subcomplex through NEAT1_2-dependent sequestration of NONO in paraspeckles.

### NEAT1_2 mediates mTORC1 regulation of NONO-dependent RNA splicing

**Figure 5:**
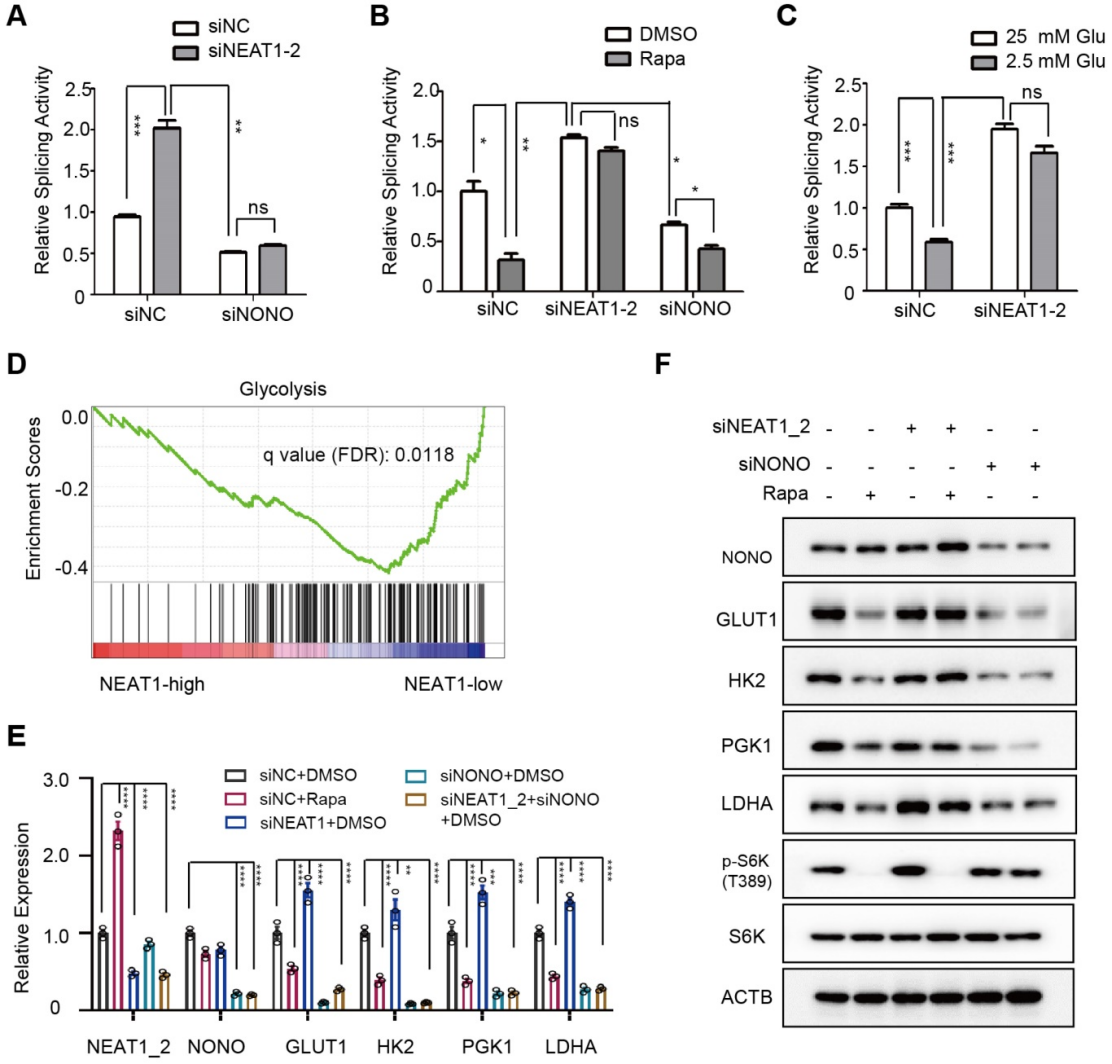
** mTORC1-NEAT1-NONO axis regulates mRNA splicing and expression of glycolytic enzymes. (A)** NEAT1_2 negatively regulates RNA splicing activity in a NONO-dependent manner. SNU423 cells were transfected with pTN24 splicing luciferase reporter and without or with NEAT1_2 knockdown in the absence or presence of NONO siRNA for 48 h. Activity of RNA splicing reporter was measured by luciferase activity, which was then normalized against the β-galactosidase reporter. mean ± SEM (n = 3) and p value was determined by unpaired two-tail Student's *t* test. *** *p* < 0.001, ** *p* < 0.01. **(B)** mTORC1 positively regulates RNA splicing activity in a NEAT1_2- and NONO-dependent manner. Activity of the luciferase splicing reporter was measured in SNU423 cells treated with 100 nM rapamycin for 24 h in the absence or presence of NEAT1_2 or NONO knockdown. β-galactosidase reporter was used for normalization. mean ± SEM (n = 3). *p* value was determined by unpaired two-tail Student's *t* test. ** *p* < 0.01, * *p* < 0.05. **(C)** Glucose stimulates RNA splicing in a NEAT1_2-dependent manner. Activity of the luciferase splicing reporter was measured in SNU423 cells cultured in high (25 mM) or low (2.5 mM) glucose for 24 h. β-galactosidase reporter was used for normalization. Data represents mean ± SEM (n = 3). *p* value was determined by unpaired two-tail Student's *t* test. ** *p* < 0.01, * *p* < 0.05.*** *p* < 0.001; ns, not significant. **(D)** Glycolysis genes are enriched in low NEAT1 expressing human primary HCC tumors. Gene enrichment analysis (GSEA) was performed in high and low NEAT1 expressing human primary HCC tumors from TCGA hepatocellular carcinoma (LIHC) transcriptome dataset. **(E)** mTORC1 positively regulates mRNA expression of glycolysis genes through NEAT1 and NONO. SNU423 cells were treated with 100 nM rapamycin for 16 h in the presence or absence of NEAT1 and/or NONO knockdown. mRNA expression of different glycolytic genes was analyzed by qRT-PCR and values are normalized against ACTIN. Data are shown as mean ± SEM (n = 3); Statistical significance was tested using two-tailed unpaired Student's *t* test. **** *p* < 0.0001. **(F)** mTORC1-NEAT1-NONO axis regulates expression of glycolytic enzyme proteins. SNU423 cells transfected with NEAT1 siRNA or a control siRNA were treated with 100 nM rapamycin for 16 h. Expression of 4 glycolytic proteins and mTORC1 signaling were analyzed by immunoblot. ACTB was used as a loading control.

mTORC1 regulation of NONO interaction with U5 spliceosome suggested a role of mTORC1 in pre-mRNA splicing. To test this, we assayed splicing activity in HCC cells using an intron-containing luciferase splicing reporter that also expresses a beta-galactosidase for normalization 47. In this assay, removal of the intron through RNA splicing of a luciferase transcript activates luciferase, enabling quantitative measurement of pre-mRNA splicing activity. Indeed, knockdown of NEAT1_2 markedly increased luciferase reporter activity in a NONO-dependent manner (Figure 5A). In contrast, rapamycin treatment strongly inhibited splicing activity, and this inhibition was blunted by NEAT1_2 knockdown (Figure 5B). Similarly, RNA splicing activity was notably higher under high glucose condition than low glucose condition, and this difference was largely eliminated after NEAT1_2 knockdown (Figure 5C). These results document that mTORC1 positively regulates RNA splicing through the NEAT1-NONO axis in response to nutrient conditions. Of note, rapamycin retained slight inhibition of splicing in NONO knockdown cells, albeit to a much smaller extent (Figure 5B), suggesting that there exists a NONO-independent mechanism for mTORC1 to regulate splicing.

### NEAT1_2 mediates mTORC1 regulation of pre-mRNA splicing and expression of key glycolytic enzymes

To ask what cellular process is regulated by NEAT1, we performed analysis of pathways that correlate with NEAT1 expression using TCGA RNA-seq data from 373 primary human HCC tumors. Glycolytic genes were found to be enriched in HCC tumors with low NEAT1 expression (Figure 5D). Specifically, NEAT1 was negatively correlated with that of key glycolytic genes GLUT1 (glucose transporter type 1), HK2 (hexokinase 2), PGK1 (phosphoglycerate kinase 1), and LDHA (lactate dehydrogenase A) (Figure S7A). Indeed, rapamycin inhibited mRNA and protein expression of GLUT1, HK2, TGK1 and LDHA, and NEAT1_2 knockdown abrogated the rapamycin inhibition (Figure 5E-F). On the other hand, NONO knockdown blocked expression of these genes regardless of rapamycin treatment or NEAT1_2 knockdown (Figure 5E-F, Figure S7B). NEAT1_2 knockdown does not affect HK2 and LDHA promoter activity (Figure S7C), indicating that mTORC1-NEAT1-NONO axis regulates several key glycolytic enzymes through a post-transcriptional mechanism.

**Figure 6:**
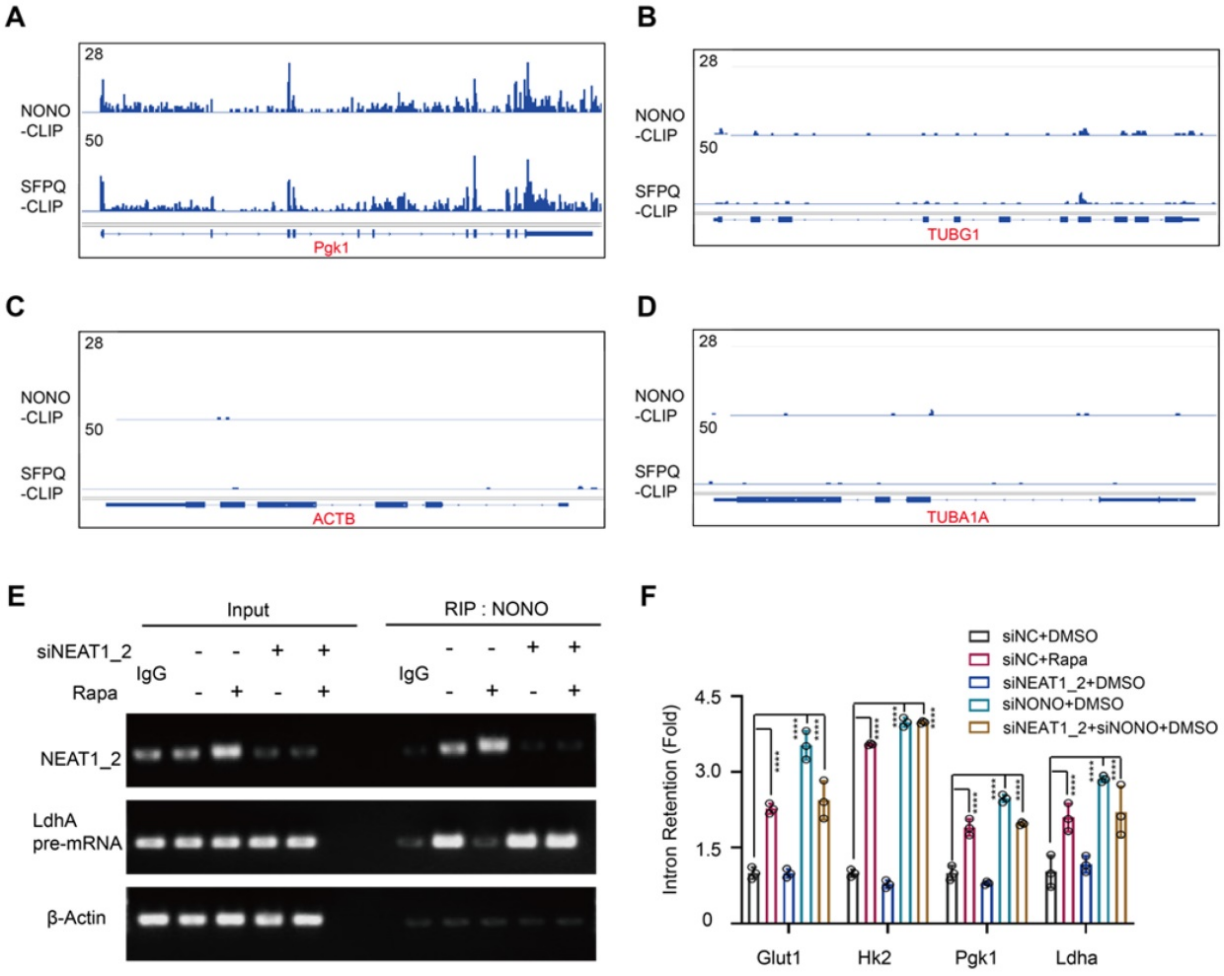
** mTORC1-NEAT1 regulates binding of NONO/SFPQ to and splicing of glycolytic pre-mRNAs. (A and B)** Binding profiles of NONO and SFPQ to LDHA and PGK1 pre-mRNA transcripts as revealed by anti-NONO and anti-SFPQ CLIP-seq. Bottom graph shows the intron-exon organization of LDHA and PGK1 pre-mRNA transcripts. **(C and D)** NONO and SFPQ do not bind to ACTB and TUBA1A pre-mRNA transcripts as revealed by anti-NONO and anti-SFPQ CLIP-seq. Bottom graph shows the intron-exon organization of ACTB and TUBA1A transcripts. **(E)** Rapamycin inhibits NONO association with the splicing junction of LDHA pre-mRNA in a NEAT1_2-dependent manner. SNU423 cells were treated with 100 nM rapamycin for 24 h in the presence or absence of NEAT1_2 knockdown. NONO was immunoprecipitated and NONO-associated LDHA pre-mRNA and NEAT1_2 was analyzed by qRT-PCR. β-ACTIN was used as negative control. **(F)** mTORC1 positively regulates splicing of glycolytic pre-mRNAs through NEAT1 and NONO. SNU423 cells were treated with 100 nM rapamycin for 16 h in the presence or absence of NEAT1 and/or NONO knockdown. Intron retention of glycolytic RNA transcripts was analyzed by qRT-PCR. Intron retention is calculated by the ratio of (Expression of intron-included region) to (Expression of intron-excluded region). Data are shown as mean ± SEM (n = 3); Statistical significance was tested using two-tailed unpaired Student's *t* test. **** *p* < 0.0001.

Glycolytic pre-mRNAs were found enriched with NONO binding motifs AGGGA 48 (Table S2), which further explains why this pathway is targeted by the NEAT1-NONO pathway. Cross-linking immunoprecipitation (CLIP) coupled with deep sequencing (CLIP-seq) is a technique for studying protein binding to RNA transcripts 49. Analysis of a global RNA transcript-binding dataset 28 showed binding peaks of NONO and SFPQ at exon-intron junctions of PGK1 and LDHA pre-mRNAs (Figure 6A-B). In contrast, no significant NONO-binding peaks were seen with ACTB and TUBA1A pre-mRNAs (Figure 6C-D), demonstrating the specificity of the CLIP-seq results. We further performed an RNA immunoprecipitation (RIP) assay. The result showed that NONO was normally associated with LDHA pre-mRNA in HCC cells, which was inhibited by rapamycin (Figure 6E). Moreover, NEAT1_2 knockdown abrogated the rapamycin effect (Figure 6E). To further interrogate the role of mTORC1 in glycolytic mRNA splicing, we analyzed unspliced glycolytic transcripts before and after rapamycin treatment using pre-mRNA-specific PCR primers that flank exon-intron junctions. Rapamycin treatment drastically increased intron retention of glycolytic pre-mRNA transcripts, (Figure 6F). While rapamycin-induced intron retention was abrogated by NEAT1_2 knockdown (Figure 6F), NONO knockdown led to elevated intron retention that was not further enhanced by rapamycin (Figure 6F). These results show that NEAT1-NONO mediates mTORC1 regulation of pre-mRNA splicing of several key glycolytic enzymes.

### mTORC1-NEAT1 signaling regulates aerobic glycolysis in HCC cells, which is important for rapamycin's action

**Figure 7:**
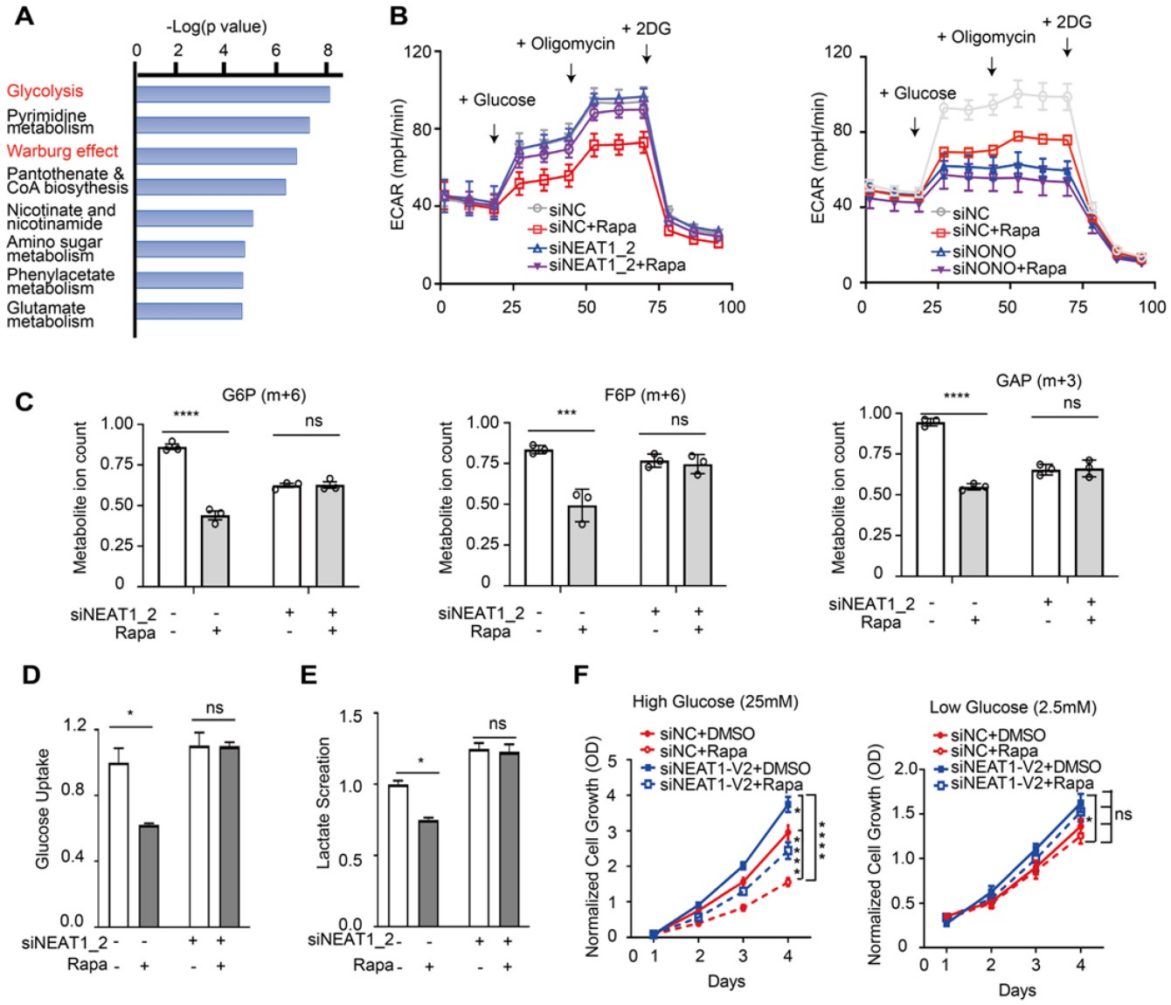
** mTORC1-NEAT1 signaling regulates aerobic glycolysis in HCC cells, which is important for rapamycin action. (A)** Metabolic pathways negatively correlated with NEAT1 expression. The overrepresentation analysis (ORA) is used to determine the significance of metabolic pathways. Metabolites Set Enrichment Analysis (MSEA) are used to evaluate the correlation of NEAT1 expression with metabolites levels. The mRNA expression and metabolomic dataset of primary liver cancer cells referenced during the study are downloaded from Cancer Cell Line Encyclopedia (CCLE) portal [https://portals.broadinstitute.org/ccle/data]. **(B)** Left panel: Rapamycin inhibits aerobic glycolysis in a NEAT1_2-dependent manner. SNU423 cells with or without NEAT1_2 knockdown were treated with 100 nM rapamycin. ECAR was analyzed by Seahorse XF Analyzer. Kinetic ECAR response of HCC cells to glucose (10 mM), oligomycin (1 µM) and 2-DG (50 mM), respectively. Each data point represents mean ± SEM, n = 4. Right panel: NONO is required for mTORC1 to promote aerobic glycolysis. SNU423 cells with or without NONO knockdown were treated with 100 nM rapamycin. ECAR was analyzed by Seahorse XF Analyzer. Kinetic ECAR response of HCC cells to glucose (10 mM), oligomycin (1 µM) and 2-DG (50 mM), respectively. Each data point represents mean ± SEM, n = 4. **(C)** mTORC1 regulates glycolytic flux in a NEAT1_2-dependent manner. SNU423 cells with or without NEAT1_2 knockdown were treated with 100 nM rapamycin, and labeled with 25 mM [U-13C]-glucose for 15 min. Glycolytic metabolites were analyzed by mass spectrometry. Shown are the ion counts for G6P (m+6), F6P (m+6) and GAP (m+3) relative to untreated control. Data (mean ± SEM, n = 3) was tested using two-tailed unpaired Student's *t* test. **** *p* < 0.0001. ns, not significant. **(D and E)** mTORC1 regulates glucose uptake and lactate secretion in a NEAT1_2-dependent manner in HCC cells. SNU423 cells with or without NEAT1_2 knockdown were treated with 100 nM rapamycin for 16 h and measured for glucose uptake (D) or lactate secretion (E). Data was normalized to cell number and presented as mean ± SEM and analyzed by unpaired two-tail Student's *t* test; * *p* < 0.05; ns, not significant. **(F)** Rapamycin significantly affects HCC cell growth under high glucose, not low glucose, culture condition. SNU423 cells with or without NEAT1_2 knockdown were cultured in high (25 mM) (I) or low (2.5 mM) (J) glucose medium and treated with 100 nM rapamycin. Cell growth was measured daily by SRB assay. Data (mean ± SEM, n = 3) were analyzed by Repeated measures ANOVA followed by Tukey's honest significance test; **** *p* < 0.0001; * *p* < 0.05, ns, not significant.

GLUT1, HK2, TGK1, and LDHA are key components of the glycolysis pathway and play key roles in cancer metabolism (Figure S8), suggesting that mTORC1-NEAT1 signaling regulates glucose metabolism. Consistently, analysis of the Cancer Cell Line Encyclopedia (CCLE) metabolomic dataset 50 of HCC cell lines showed that glycolysis and Warburg effect pathways were up-regulated in HCC cells exhibiting low NEAT1 expression (Figure 7A). To test this hypothesis, we measured the rate of glycolysis in Huh1 cells under different conditions by ECAR using Seahorse-XF Analyzer. Our results showed rapamycin inhibition of glycolysis, which was abrogated by NEAT1_2 knockdown (left panel, Figure 7B). Conversely, knockdown of NONO was sufficient to blunt glycolysis in the ECAR assay regardless of rapamycin treatment (right panel, Figure 7B). [U-^13^C]-glucose labeling and metabolomic analysis of HCC cells showed that rapamycin attenuated glycolytic flux as indicated by reduced level of ^13^C-labeled G6P (m+6), F6P (m+6) and GAP (m+3) (Figure 7C). Rapamycin no longer inhibited glycolytic flux after NEAT1_2 knockdown (Figure 7C).

Because rapamycin inhibits splicing of glucose transporter type 1 (GLUT1) and lactate dehydrogenase A (LDHA), we further measured glucose uptake and lactate secretion, two signature events of aerobic glycolysis 51, 52. Rapamycin suppressed both glucose uptake and lactate secretion in a NEAT1_2-dependent (Figure 7D-E). Because rapamycin also induced NEAT1_1 expression, we investigated the effect of ectopic NEAT1_1 expression. However, NEAT1_1 overexpression did not significantly affect glucose uptake/lactate secretion, or NEAT1_2 knockdown-induced change in glucose uptake/lactate secretion (Figure S9A-D). Aerobic glycolysis plays an essential role in supporting tumor growth. We hence hypothesized that the mTORC1-NEAT1 axis plays a role in liver cancer cell growth and rapamycin's action. To test this, we examined the antiproliferative activity of rapamycin on HCC cells under high and low glucose culture conditions. Rapamycin displayed strong growth inhibition of HCC cells under high glucose condition, which was attenuated by NEAT1_2 knockdown (left panel , Figure 7F). In contrast, neither rapamycin nor NEAT1_2 significantly affected HCC growth under low glucose conditions, albeit the overall growth rate was also lower due to limited nutrition (right panel, Figure 7F), suggesting that mTORC1-NEAT1 axis promotes rapamycin-sensitive cancer cell growth through glucose metabolism.

### NEAT1_2 restrains AKT-mTORC1-mediated aerobic glycolysis and liver tumor development *in vivo*

**Figure 8:**
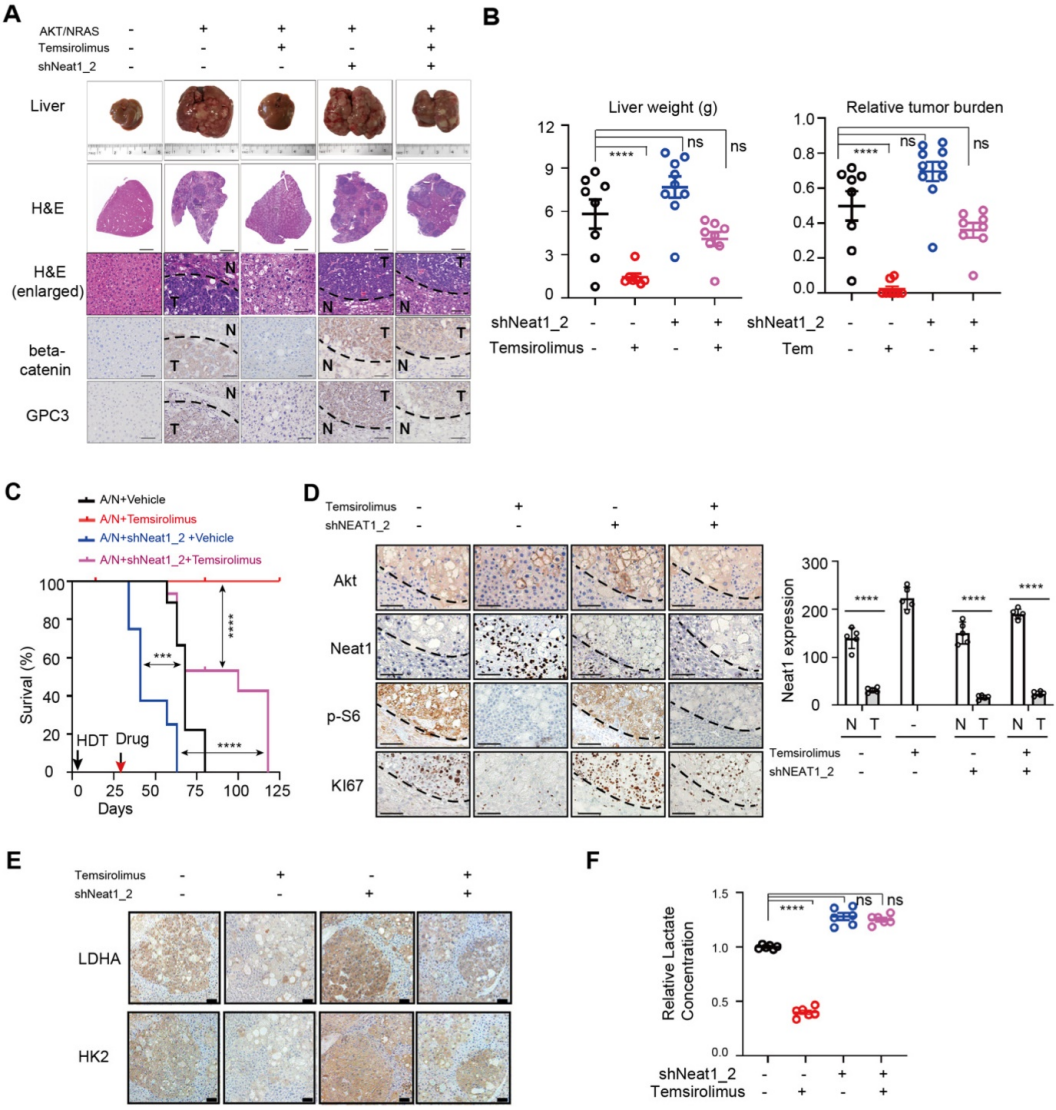
** NEAT1 is important for mTORC1-targeted therapeutic response *in vivo*. (A)** Representative images of liver and liver tissues with H&E staining and IHC staining with HCC markers beta-catenin and glypican 3 (GPC3) from HDT mouse liver tumor models (mAKT/NRAS +/- shNEAT1_2) treated with temsirolimus or a drug vehicle. Liver tissues were collected at 63 days post HDT. **(B)** Liver weight (left panel) and relative tumor burden (right panel) from 4 different animal groups (AKT/NRAS/Vehicle, n = 8; AKT/NRAS/temsirolimus, n = 7; AKT/NRAS/shNEAT1/Vehicle, n = 9; AKT/NRAS/shNEAT1/temsirolimus, n = 8). *p* value was determined by one-way ANOVA followed by Tukey's multiple comparisons test. **** *p* < 0.0001. ns, not significant. **(C)** Kaplan‐Meier survival analysis of different animal groups (AKT/NRAS/Vehicle, n = 9; AKT/NRAS/temsirolimus, n = 10; AKT/NRAS/shNEAT1/Vehicle, n = 8; AKT/NRAS/shNEAT1/temsirolimus, n = 7). *** *p* < 0.001, **** *p* < 0.0001. **(D)** Representative IHC staining for AKT, NEAT1, p-S6 and Ki67 from liver tumor tissues (mAKT/NRAS +/- shNEAT1_2 treated with temsirolimus or a drug vehicle). Scale bar, 100 µm. **(E)** Protein levels of LDHA and HK2 as determined by IHC analysis from different liver tumor tissues (mAKT/NRAS +/- shNEAT1_2 treated with temsirolimus or a drug vehicle). Scale bar, 100 µm. **(F)** Temsirolimus inhibits glycolysis of AKT/NRAS-driven liver tumors in a NEAT1_2-dependent manner. Lactate concentration in liver tumors tissues (mAKT/NRAS +/- shNEAT1_2 treated with temsirolimus or a drug vehicle) was measured using a colorimetric lactate assay kit. Data was normalized and Results represent mean ± SEM (n = 6). *p* value was determined by one-way ANOVA followed by Tukey's multiple comparisons test. **** *p* < 0.0001. ns, not significant.

To evaluate the role of NEAT1 in mTORC1-dependent aerobic glycolysis, liver tumor development and therapy *in vivo*, we generated mAKT/NRAS-driven mouse liver cancer by hydrodynamic transfection (HDT) in the absence or presence of shNeat1_2 (Figure S10). After liver tumors were established (26 days post HDT), mice were treated with temsirolimus (or drug vehicle). The drug vehicle control mAKT/NRAS group developed HCC tumors as judged by H&E staining, histology and IHC staining with tumor markers beta-Catenin and Gpc3 (Figure 8A-B), with a mean survival time of 70 days (Figure 8C). Temsirolimus treatment blunted tumor development and extended survival of virtually all treated animals beyond the endpoint of the study (125 days) (Figure 8A-C). Neat1_2 knockdown accelerated mAKT/NRAS-driven tumor development and shortened the mean survival time to 40 days (Figure 8A-C), which was likely due to further reduction of Neat1_2 expression (Figure 8D). Moreover, mAKT/NRAS+shNeat1_2 tumors were refractory to temsirolimus treatment compared to the mAKT/NRAS group (Figure 8A-C).

At the molecular level, temsirolimus abolished p-S6 in both mAKT/NRAS and mAKT/NRAS+shNeat1_2 tumors, indicating that the drug achieved on-target mTORC1 inhibition in both groups (Figure 8D). As seen *in vitro*, temsirolimus stimulated Neat1_2 expression in liver tumors, which was correlated with attenuated tumor cell proliferation as judged by Ki67 staining (Figure 8D). However, temsirolimus's antitumor effect was attenuated in the mAKT/NRAS+shNeat1_2 group despite on target mTORC1 inhibition (Figure 8D). HK2 and LDHA proteins were overexpressed in mAKT/NRAS-driven tumors compared with adjacent normal liver tissues, and their overexpression was suppressed by temsirolimus (Figure 8E). However, temsirolimus inhibited HK2 and LDHA overexpression to a lesser extent in the Neat1_2 knockdown tumors (Figure 8E). Consistently, temsirolimus treatment markedly decreased the lactate level in mAKT/NRAS tumors, but not mAKT/NRAS/shNeat1_2 tumors (Figure 8F). Together, these observations indicate that Neat1_2 is important for restraining mTORC1 promotion of aerobic glycolysis and liver tumor development, which is important for response to mTORC1-targeted therapy.

## Discussion

Metabolic reprogramming from oxidative phosphorylation to aerobic glycolysis is a hallmark of cancer, a phenomenon called “Warburg Effect” that is crucial to support rapid cancer cell growth, survival, and invasiveness 19. mTORC1 signaling is commonly activated in HCC, driving liver tumorigenesis. Although mTORC1 has been recognized as a major regulator of Warburg Effect in cancer, the underlying mechanism remains not well understood. For example, mTORC1 was shown to regulate anaerobic glycolysis through the HIF1α pathway in mouse embryonic fibroblasts 20, 21. However, HIF1α is essentially undetectable in HCC cells under normal oxygen or normoxic condition (Figure S11), indicating that HIF1α-dependent mechanism is not involved under aerobic condition in this context. Upon oncogenic activation during liver tumorigenesis, mTORC1 represses *NEAT1_2* transcription and paraspeckle biogenesis, resulting in up-regulation of aerobic glycolysis to meet the anabolic and energetic demand of uncontrolled cancer cell growth. Our demonstration that mTORC1 regulates aerobic glycolysis is in contrast to the HIF1α-dependent regulation of hypoxic glycolysis shown in the aforementioned studies, which underscores distinct mechanisms employed by mTORC1 under different oxygen conditions in tumor microenvironments.

Rapalogs, such as temsirolimus, are US FDA-approved oncology therapeutics 53. However, previous human clinical trials of rapalogs in advanced HCC failed to achieve desired endpoints and better patient survival outcomes 15, 16. The mechanism of response and resistance to mTOR-targeted therapies in HCC remains poorly understood. We showed that rapamycin and temsirolimus cause a robust up-regulation of NEAT1_2 and paraspeckle biogenesis in HCC. Importantly, down-regulation of NEAT1_2 renders resistance of HCC cells to rapamycin and temsirolimus *in vitro* and *in vivo* by sustaining aerobic glycolysis and oncogenic growth. Interestingly, rapamycin inhibition of HCC cell growth is strongly dependent on high glucose.

Many cancer cells are deprived of nutrients in poorly angiogenic tumor microenvironments, a condition likely to limit rapalog efficacy that may explain the failed clinical trials. Collectively, our observations reveal that NEAT1 expression/paraspeckle biogenesis is a key determinant for the success of mTORC1-targeted cancer therapy in liver cancer. It should be noted that the anticancer effect of mTORC1 inhibition in HCC is context-dependent. For example, in obesity-promoted HCC or HCC deficient of autophagy in mice, rapamycin treatment has been reported to cause liver damage and exacerbate hepatopathogenesis 54, 55. Further studying mTORC1-NEAT1 axis in response to rapamycin treatment under these conditions could help better understand mTOR's complex role in liver pathogenesis and help guide precision therapy.

mTOR is known to regulate expression of rRNAs, tRNAs, and certain mRNA-encoding genes 26. Herein, we showed that mTORC1 also regulates lncRNA transcriptome. We focused on characterizing one of mTOR target lncRNAs, NEAT1. *NEAT1* gene encodes two completely overlapping NEAT1 isoforms: NEAT1_1 and NEAT1_2. Although mTORC1 regulates both isoforms, this study was mainly focused on NEAT1_2 because low expression of NEAT1_2, not NEAT1_1 is significantly associated with poor overall survival of HCC patients. Moreover, our data indicate that NEAT1_2, rather than NEAT1_1 mediates mTORC1 signaling to controls aerobic glycolysis in HCC cells. Mechanistically, through regulation of *NEAT1_2*, mTORC1 negatively controls paraspeckle biogenesis. Aberrant mRNA splicing is a major contributor to pathologic processes, such as tumorigenesis 56, 57. Upon oncogenic activation, mTORC1 restrains NEAT1_2 expression and paraspeckle biogenesis, liberating NONO/SFPQ from paraspeckles. Strikingly, glycolytic pre-mRNAs are enriched in NONO/SFPQ consensus binding motifs (Table S2), providing a molecular basis for NONO/SFPQ to recognize these pre-mRNAs and recruitment of U5 spliceosome, enhancing pre-mRNA splicing and expression of several key glycolytic genes. This mechanism enables oncogenic mTORC1 to promote aerobic glycolysis and HCC growth and development.

## Supplementary Material

Supplementary figures and tables.

## Abbreviations

HCC: Hepatocellular Carcinoma
mTOR): Mechanistic target of rapamycin
lncRNA: long non-coding RNA
NEAT1: Nuclear Paraspeckle Assembly Transcript 1
RRE: Rapamycin Response Element
S6k: Ribosomal protein S6 kinase
MEF: Mouse Embryonic Fibroblasts
GLUT1: Glucose transporter type 1
HK2: hexokinase 2
PGK1: Phosphoglycerate Kinase 1
LDHA: Lactate Dehydrogenase A
HDT: hydrodynamic transfection
H&E staining: hematoxylin and eosin staining
IHC: Immunohistochemistry
PLA: Proximity ligation assays
GEPIA: Gene Expression Profiling Interactive Analysis
