# Supplementary material for: mTOR regulates aerobic glycolysis through NEAT1 and nuclear paraspeckle-mediated mechanism in hepatocellular carcinoma
Source: Theranostics. 2022 Apr 24;12(7):3518–33. doi: 10.7150/thno.72581 (PMC9065186; doi:10.7150/thno.72581)
Supplement: Supplementary file 1 — Supplementary figures and tables. [file thnov12p3518s1.pdf]

Supplementary Information

mTOR regulates aerobic glycolysis through NEAT1 and nuclear paraspeckle-mediated mechanism in hepatocellular carcinoma

Hong Zhang, Xiaoyang Su, Stephen K. Burley and X.F. Steven Zheng*

*Corresponding author. Email: zhengst@cinj.rutgers.edu.

Table of Contents

Figure S1-S11 with figure legends

Tables S1-S6

Supplementary Materials and Methods

SUPPLEMENTARY FIGURES

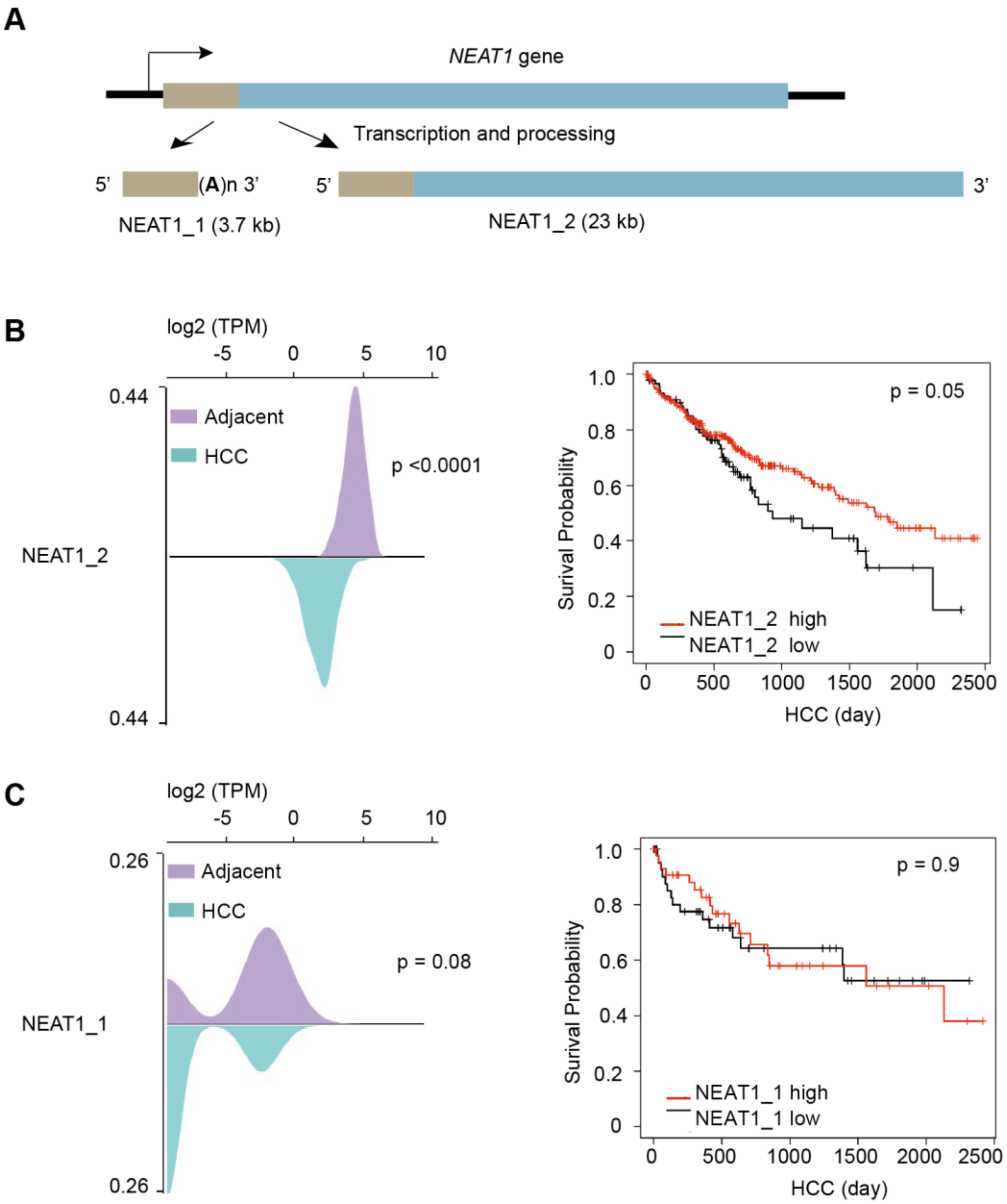

Figure S1. **NEAT1_2 may function dominantly during HCC progression.**

(**A**) The human *NEAT1* encodes two completely overlapping NEAT1 isoforms, NEAT1_1 and NEAT1_2. NEAT1_1 is polyadenylated while NEAT1_2 is not. (**B**, **C**) The expression and density of NEAT1_2 **(B)** and NEAT1_1 (**C**) transcripts were analyzed in 369 primary HCC and 160 normal liver tissues. The sample information and mRNA expression data of NEAT1_2 and NEAT1_1 transcript was analyzed and downloaded from UCSC Xena portal (https://xenabrowser.net/). p value was determined by unpaired two-

tailed Student's t test (Left panel). HCC patients with low NEAT1 expression have poor overall survival. (Right panel) Kaplan-Meier analysis was used to compare overall survival of HCC patients with high and low NEAT1_2 (**B**) or NEAT1_1 (**C**) expression. p value was calculated using the two-sided log-rank test.

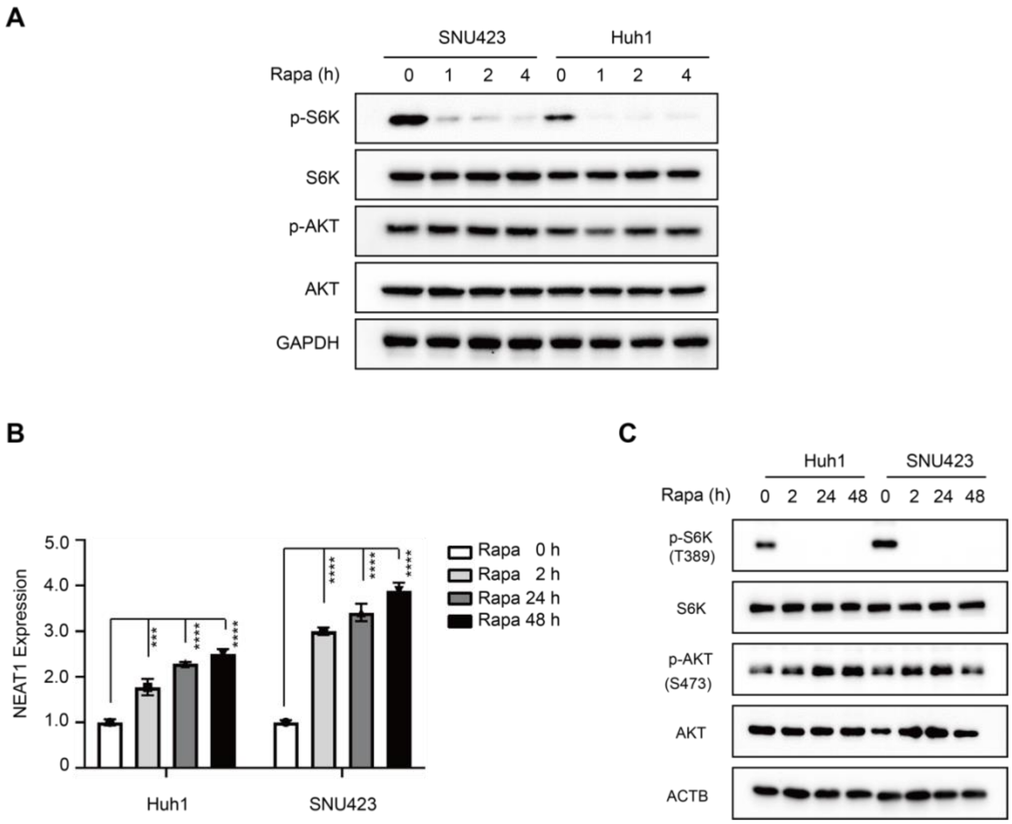

Figure S2. **Acute and chronic rapamycin treatment inhibits mTORC1 signaling and induces NEAT1 expression in HCC cells.**

(**A**) SNU423 and Huh1 cells were treated with 100 nM rapamycin for 0, 1, 2 and 4 h. Rapamycin effect on mTORC1 and mTORC2 signaling was determined by immunoblot of p-S6K(T389) and p-AKT(S473), respectively. GAPDH was used as a loading control. (**B**, **C**) SNU423 and Huh1 cells were treated with 100 nM rapamycin for 0, 2, 24 and 48 h. NEAT1 expression was analyzed by qRT-PCR and values are normalized against ACTIN (B). Data are shown as mean ± SEM (n = 3); Statistical significance was tested using one-way ANOVA followed by Tukey's multiple comparisons test. **** $p < 0.0001$. The effect of rapamycin on mTORC1 and mTORC2 signaling was determined by immunoblot of p-S6K(T389) and p-AKT(S473), respectively. ACTB was used as a loading control (**C**).

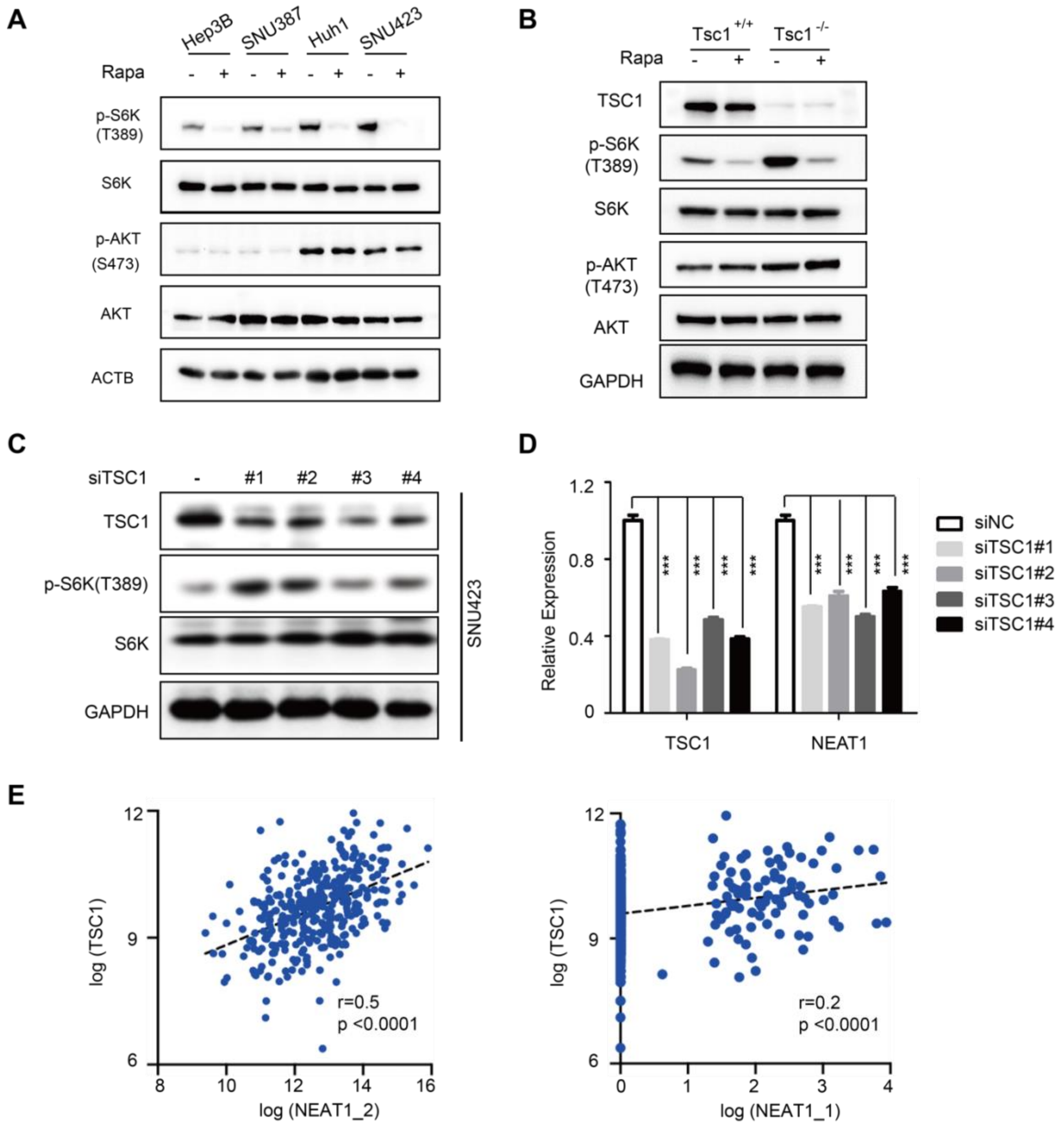

Figure S3. Activation of mTORC1 signaling by TSC1 down-regulation suppresses NEAT1 expression.

(**A**) Effect of rapamycin on mTOR signaling in different HCC cell lines. Hep3B, SNU387, Huh1 and SNU423 cells were treated without or with 100 nM rapamycin. mTOR signaling was determined by immunoblot of p-S6K(T389), S6K, p-AKT(S473) and AKT, respectively. ACTB was used as a loading control. (**B**) Effect of rapamycin on mTOR signaling in MEF-wild type and MEF-*Tsc1*$^{-/-}$ cells. WT and mutant MEF cells were treated without or with 100 nM rapamycin for 2 h and analyzed for mTOR signaling

by immunoblot. (**C**) SNU423 cells were transfected with 4 independent TSC1 siRNAs and measured for mTORC1 activation by pS6K1. GAPDH was used as a loading control. (**D**) NEAT1 expression in control and TSC1 knockdown cells was measured by qRT-PCR. Relative NEAT1 expression was calculated relative to Actin. Data represent mean ± SEM (n = 3). p value was determined by one-way ANOVA followed by Tukey's multiple comparisons test. *** $p < 0.001$. (**E**) NEAT1_2 expression is negatively correlated with mTORC1 signaling in human HCC tumors. Correlation analysis of TSC1 mRNA expression with NEAT1_2 RNA (left panel) or NEAT1_1 RNA (right panel) in 369 primary human HCC tumors. p value was analyzed by nonparametric Spearman correlation test.

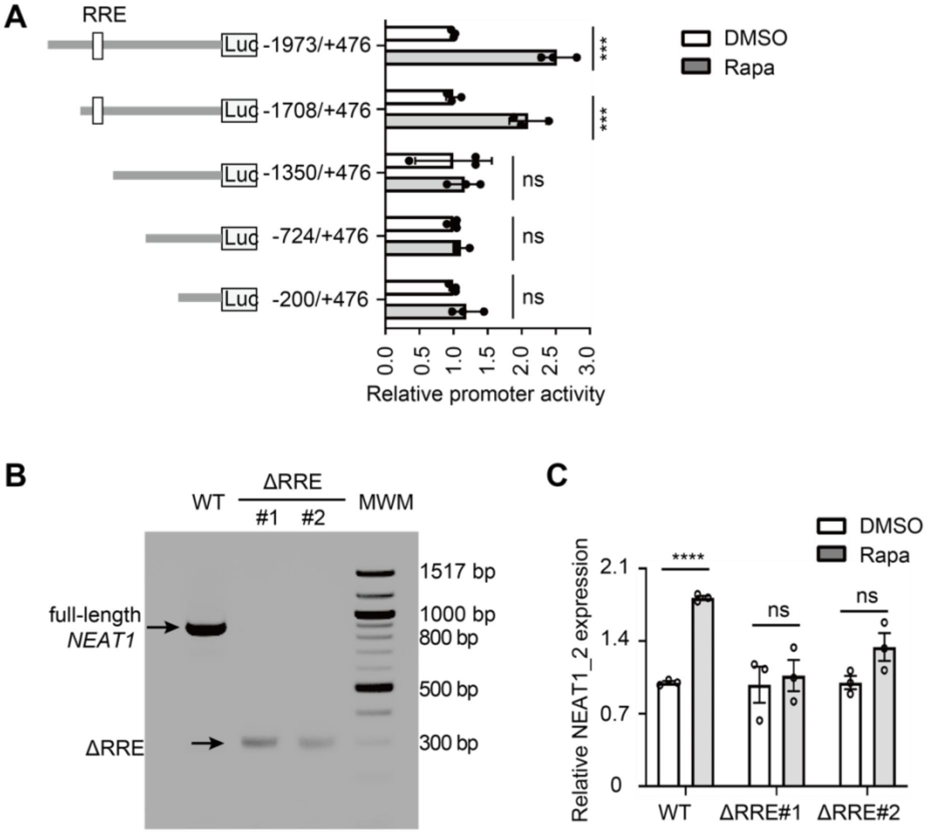

Figure S4. mTORC1 acts at NEAT1 promoter and negatively regulates NEAT1 transcription.

(**A**) Sequential deletion analysis of NEAT1 promoter as assayed by luciferase reporter in SNU423 cells in the absence or presence of 100 nM rapamycin. Results were normalized to the DMSO control within each group. Mean ± SEM (n = 3), unpaired two-tail t test; *** $p < 0.001$. (**B**) PCR analysis of the Rapamycin Response Region (RRE) genomic DNA region in wild type and RREΔ SNU423 cells. MWM, DNA molecular weight marker. (**C**) CRISPR/cas9-mediated deletion of RRE in SNU423 cells blunts rapamycin induction of NEAT1 expression. WT and two independent clones of RRE genomic deletion SNU423 cells were treated with 100 nM rapamycin or drug vehicle (DMSO) for 2 h. NEAT1 expression was analyzed by RT-qPCR. Results were normalized to NEAT1 expression of normal condition within each cell type. Mean ± SEM (n = 3), unpaired two-tail t test; **** $p < 0.0001$. ns, not significant.

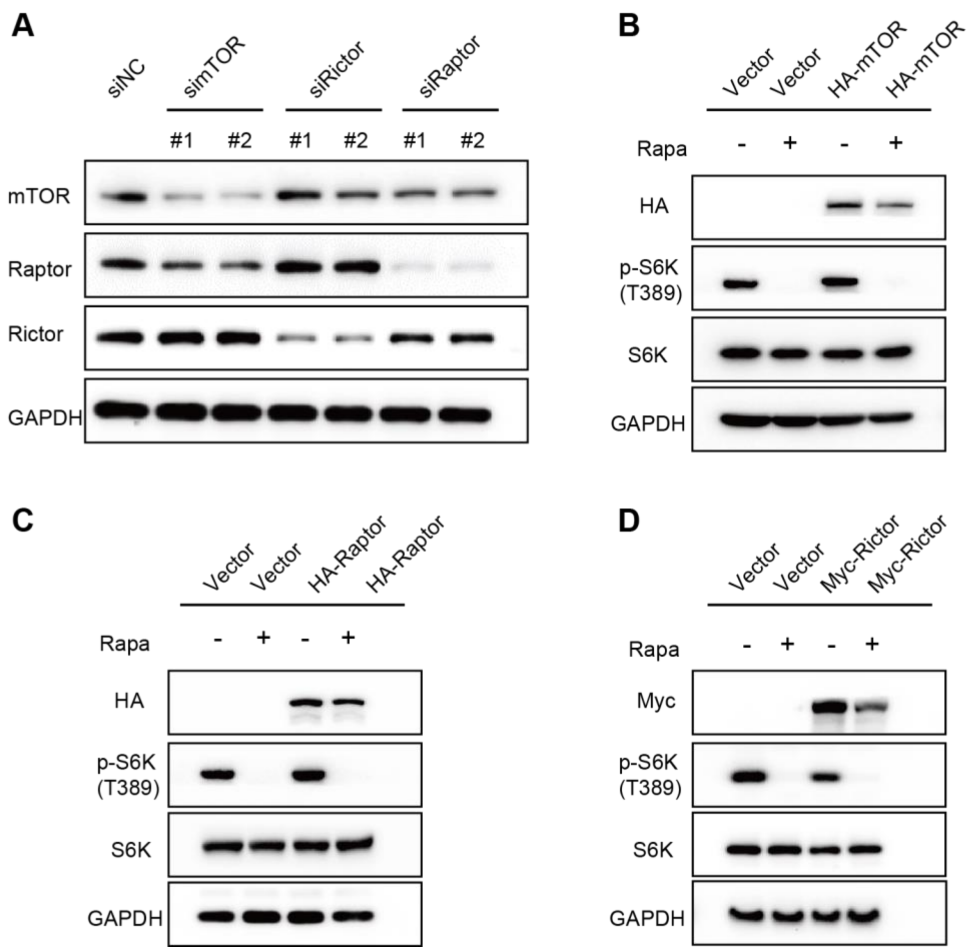

Figure S5. Expression of si-mTOR, HA-mTOR, HA-Raptor and Myc-Rictor in HCC cells for ChIP assays. (A) SNU423 cells were transfected with 2 independent mTOR siRNAs and measured for mTOR protein expression. GAPDH was used as a loading control. **(B-D)** SNU423 cells transiently expressing HA-mTOR**(B)**, HA-Raptor **(C)** and Myc-Rictor **(D)** were treated with or without 100 nM Rapamycin for 2 h and cells were analyzed for expression of tagged proteins, and mTORC1 signaling by immunoblot of p-S6K and S6K. GAPDH was used as a loading control.

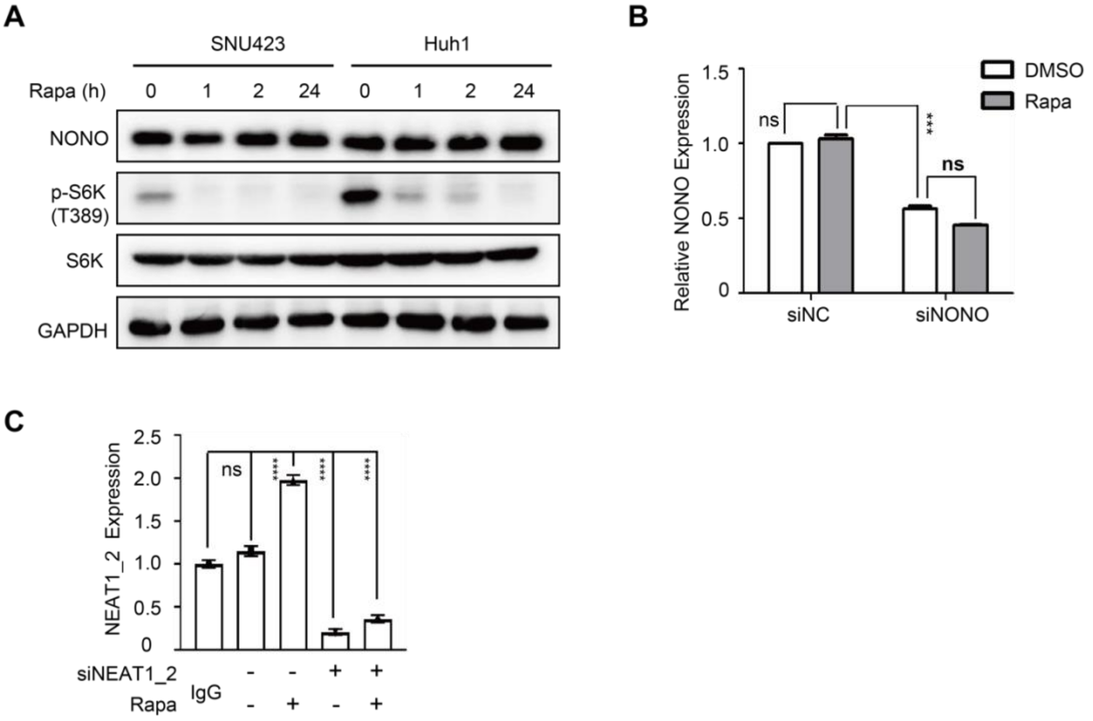

Figure S6. Rapamycin does not affect NONO protein and mRNA expression in HCC cells. (A) Rapamycin does not affect NONO protein expression in HCC cells. SNU423 and Huh1 cells were treated with 100 nM rapamycin for different times and measured for the level of NONO protein, p-S6K and S6K by immunoblot. GAPDH was used as a loading control. **(B)** Rapamycin does not affect NONO mRNA expression in HCC cells. SNU423 cells were transfected with control siRNA (siNC) or NONO siRNA (siNONO), and treated with 100 nM rapamycin for 24 h. NONO mRNA expressed was measured by qRT-PCR. Relative NONO expression was calculated relative to ACTIN. Data represent mean ± SEM (n = 3). p value was determined by one-way ANOVA followed by Tukey's multiple comparisons test. *** $p < 0.001$. ns, not significant. **(C)** Rapamycin significantly induces NEAT1_2 expression in HCC cells. SNU423 cells were transfected with control siRNA (siNC) or NEAT1_2 siRNA (siNEAT1_2), and treated with 100 nM rapamycin for 24 h. NEAT1_2 expression was measured by qRT-PCR and calculated relative to ACTIN. Data represent mean ± SEM (n = 3). p value was determined by one-way ANOVA followed by Tukey's multiple comparisons test. **** $p < 0.0001$. ns, not significant.

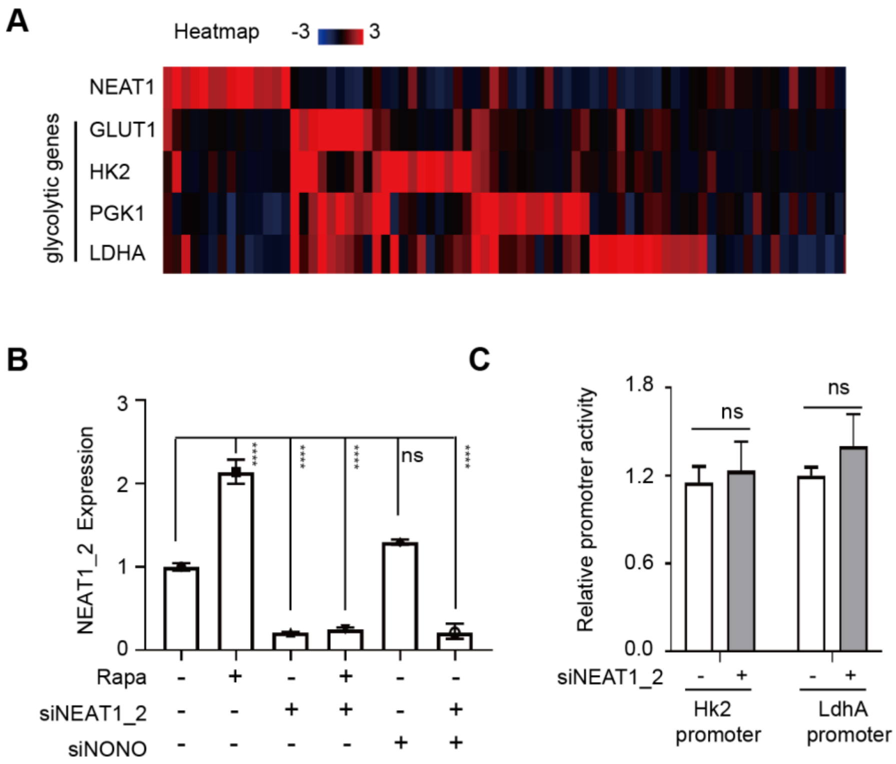

Figure S7. mTORC1-NEAT1-NONO axis regulates mRNA splicing and expression of key aerobic glycolytic enzymes. **(A)** NEAT1 expression is negatively correlated with glycolytic genes in human primary HCC tumors. Heatmap shows high NEAT1 expression is mutually exclusive from high expression of glycolytic genes GLUT1, HK2, PGK1 and LDHA in human primary HCC tumors from TCGA hepatocellular carcinoma (LIHC) transcriptome dataset. **(B)** NEAT1_2 expression under different conditions. SNU423 cells were treated with 100 nM rapamycin for 16 h in the presence or absence of NEAT1 and/or NONO knockdown. NEAT1_2 expression was analyzed by qRT-PCR and normalized against ACTIN. Data are shown as mean ± SEM (n = 3). p value was determined by one-way ANOVA followed by Tukey's multiple comparisons test. **** $p < 0.0001$. **(C)** NEAT1_2 does not regulate transcription of glycolytic genes. SNU423 cells were transfected with NEAT1_2 siRNA or a control siRNA, and measured for HK2 and LDHA promoter activity using luciferase reporter. Data represent mean ± SEM (n = 3). p value was determined by unpaired two-tailed Student's t test; ns, not significant.

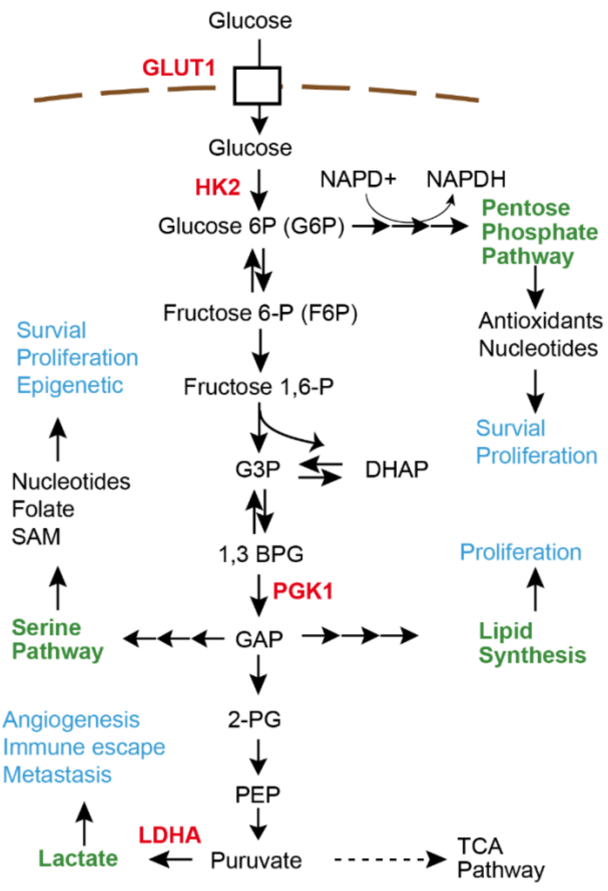

Figure S8. mTORC1-NEAT1 signaling regulates aerobic glycolysis in HCC cells, which is important for rapamycin's action. Shown is glycolysis pathway with key metabolic genes regulated by mTORC1-NEAT1 axis marked in red.

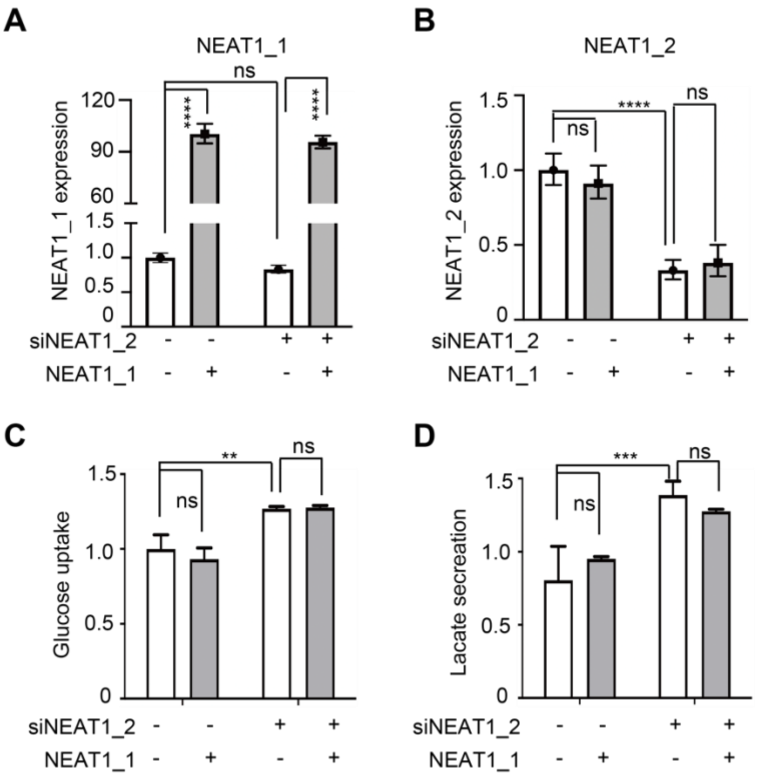

Figure S9. NEAT1_1 showed moderate effect on glucose metabolism in HCC cells. (**A**) NEAT1_1 was ectopically overexpressed in HCC cells. SNU423 cells were transfected without or with pCDNA3.1-NEAT1_1 plasmid in the absence or presence of NEAT1_2 knockdown and analyzed for NEAT1_1 expression by qRT-PCR. NEAT1_1 expression was calculated as relative to ACTIN. Bar graphs represent mean ± SEM (n = 3). Data are shown as mean ± SEM (n = 3); Statistical significance was tested using two-tailed unpaired t test. **** $p < 0.0001$, ns, not significant. (**B**) NEAT1_2 was specifically knockdown in SNU423 cells. SNU423 cells were transfected without or with pCDNA3.1-NEAT1_1 plasmid in the absence or presence of NEAT1_2 knockdown and analyzed for NEAT1_2 expression by qRT-PCR. NEAT1_2 expression was calculated as relative to ACTIN. Bar graphs represent mean ± SEM (n = 3). Data are shown as mean ± SEM (n = 3); Statistical significance was tested using two-tailed unpaired t test. **** $p < 0.0001$, ns, not significant. (**C**-**D**) NEAT1_2, not NEAT1_1 regulates glucose uptake and lactate secretion in HCC cells. SNU423 cells were transfected without or with pCDNA3.1-NEAT1_1 plasmid in the absence or presence of NEAT1_2 knockdown and measured for glucose uptake (**C)** or lactate secretion

(**D**). Data was normalized to cell number and presented as mean ± SEM and analyzed by unpaired two-tail Student's *t* test; * $p < 0.05$; ns, not significant.

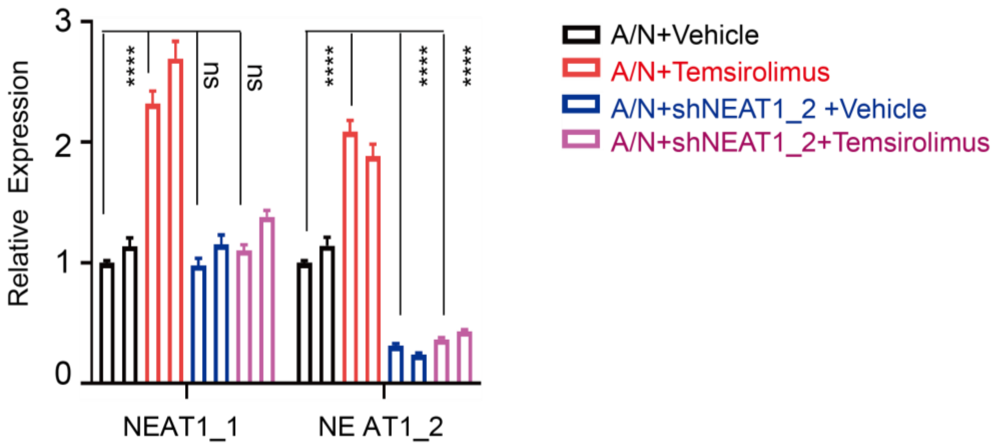

Figure S10. Expression of NEAT1_1 and NEAT1_2 in mAKT/NRAS +/- shNEAT1_2 treated without or with Temsirolimus.

HDT mouse liver tumors (mAKT/NRAS +/- shNEAT1_2) treated with temsirolimus or a drug vehicle. Liver tissues were collected at 63 days post HDT and analyzed for the expression level of NEAT1_1, NEAT1_2 by qRT-PCR. Values are normalized against Actin mRNA. Bar graphs represent mean ± SEM (n = 6). p value was determined by one-way ANOVA followed by Tukey's multiple comparisons test. *** $p < 0.001$. ns, not significant.

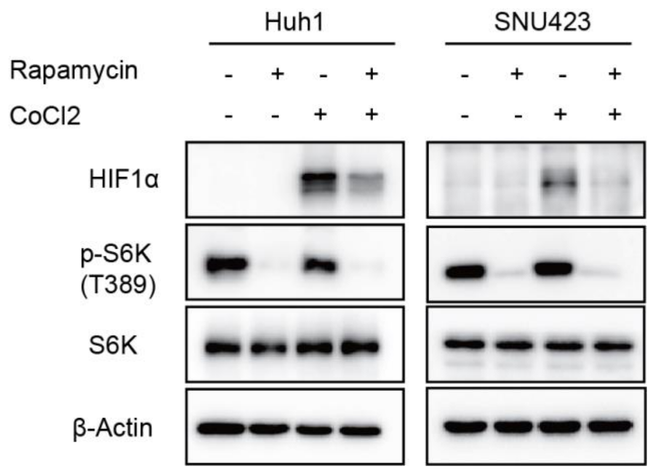

Figure S11. mTORC1 regulates HIF-1α protein expression under hypoxia, but not normoxia. Huh1 and SNU423 cells were treated without or with 100 nM rapamycin treatment for 24 h under hypoxia (+100 μM $CoCl_2$) or normoxia (-100 μM $CoCl_2$). HIF1a protein, p-S6K1 and S6K1 protein were analyzed by immunoblot. β-Actin was used as an internal control.

SUPPLEMENTARY TABLES

Table S1. Significantly Altered lncRNAs by Rapamycin in HCC

Gene_Name	RaPa_ FPKM	ctrl_ FPKM	FC (Rapa/ctrl)	p value
LURAP1L-AS1	2.11	0.46	4.59	0.01
HOXB-AS4	0.50	2.09	0.24	0.05
RP11-12J10.4	0.34	2.18	0.15	0.01
CTC-425F1.4	11.84	0.00	#DIV/0!	0.00
RP11-148B18.3	5.11	0.00	#DIV/0!	0.00
RP11-70C1.3	10.17	0.00	#DIV/0!	0.00
RP11-498C9.4	5.16	0.31	16.51	0.04
RP11-229E13.4	25775.00	1562.52	16.50	0.00
KDM4A-AS1	2.28	0.17	13.59	0.03
SNHG12	4.30	0.38	11.38	0.00
SCARNA9	143.07	15.07	9.50	0.00
CTB-12A17.3	18.08	2.10	8.62	0.00
XXbac-BPG252P9.10	4.64	0.56	8.34	0.00
RMRP	1070000.00	129244.00	8.28	0.05
CTD-2269F5.1	13.38	1.81	7.40	0.05
SNHG8	202.29	32.94	6.14	0.00
RP11-225B17.2	3.43	0.56	6.13	0.03
RP11-386I14.4	129.20	22.55	5.73	0.01
AC010761.10	12.90	2.35	5.49	0.01

PSMA3-AS1	6.62	1.21	5.48	0.00
SNHG3	404.84	78.27	5.17	0.05
EPHA5-AS1	6.69	1.38	4.84	0.00
SNHG1	12.01	2.90	4.13	0.00
AC108488.4	4.51	1.13	3.98	0.05
XXbac-B444P24.14	2.74	0.69	3.94	0.04
AC108142.1	5.64	1.44	3.92	0.00
CTC-367J11.1	5.26	1.36	3.88	0.04
RP11-140K17.3	7.82	2.05	3.82	0.02
ZNF667-AS1	2.89	0.76	3.81	0.03
APCDD1L-AS1	5.49	1.46	3.76	0.01
CH17-373J23.1	67.50	18.04	3.74	0.00
RP11-547C5.1	25.46	6.92	3.68	0.02
RP11-392P7.6	5.77	1.61	3.59	0.00
COX10-AS1	2.30	0.64	3.57	0.00
OIP5-AS1	4.16	1.17	3.57	0.01
AC002467.7	2.89	0.82	3.52	0.02
RP11-805J14.5	13.37	3.81	3.51	0.00
RP11-1148L6.8	7.86	2.31	3.40	0.01
THUMPD3-AS1	12.49	3.67	3.40	0.00
RPPH1	60389.20	17932.70	3.37	0.05
MIR17HG	3.41	1.05	3.26	0.00
LINC00702	13.63	4.26	3.20	0.00

RP11-6N17.4	30.39	9.69	3.14	0.00
RP1-261G23.7	570.50	184.75	3.09	0.05
FGD5-AS1	3.85	1.33	2.91	0.01
CTD-2269F5.1	2.64	0.92	2.87	0.02
SCARNA10	4399.26	1536.96	2.86	0.00
CRNDE	20.92	7.75	2.70	0.00
RP11-1069G10.2	3.37	1.27	2.65	0.02
LINC00998	51.01	19.38	2.63	0.00
RP11-417L19.6	21.00	7.98	2.63	0.03
ZFAS1	55.34	22.81	2.43	0.00
ZBED5-AS1	4.06	1.68	2.41	0.02
ERVK3-1	9.84	4.08	2.41	0.01
RNU12	23.12	9.75	2.37	0.00
CH507-513H4.5	5.28	2.23	2.37	0.01
SNHG6	113.29	47.91	2.36	0.00
RP4-738P15.6	72.43	31.05	2.33	0.01
CTD-2256P15.4	27.30	11.80	2.31	0.00
MAPKAPK5-AS1	4.25	1.86	2.29	0.04
TTC28-AS1	5.64	2.47	2.29	0.02
NEAT1	17.12	7.62	2.25	0.03
CTD-2545M3.2	28.85	13.20	2.19	0.02
SNHG5	187.14	86.66	2.16	0.00
CH507-513H4.4	11.83	5.49	2.15	0.01

CTD-3014M21.1	4.75	2.26	2.10	0.03
RP11-101E13.5	13.18	6.30	2.09	0.02
LRRC75A-AS1	256.30	123.47	2.08	0.00
RAB30-AS1	26.37	12.84	2.05	0.02
CH507-513H4.6	11.41	5.60	2.04	0.02
ANKRD10-IT1	10.17	5.00	2.03	0.03
RP11-394B2.1	3.17	6.30	0.50	0.01
RAET1E-AS1	9.44	18.80	0.50	0.01
STEAP3-AS1	1.86	3.71	0.50	0.02
RP11-342D11.2	7.66	15.31	0.50	0.03
RP11-1055B8.1	1.57	3.15	0.50	0.05
RP11-46D6.1	2.91	5.88	0.50	0.02
RP11-473I1.5	9.89	20.01	0.49	0.02
RP3-430N8.11	7.98	16.18	0.49	0.03
RP11-214O1.2	2.27	4.63	0.49	0.03
AC093110.3	4.95	10.23	0.48	0.04
RP11-175O19.4	2.03	4.22	0.48	0.01
RP11-366L20.2	1.44	3.00	0.48	0.01
AC009133.15	29.58	61.88	0.48	0.01
RP11-96K19.5	1.77	3.75	0.47	0.03
FRMD6-AS1	1.24	2.62	0.47	0.02
MED14OS	3.91	8.28	0.47	0.02
CTD-2033D15.3	5.79	12.28	0.47	0.03

RP11-618K13.2	10.52	22.35	0.47	0.03
LINC01578	4.39	9.34	0.47	0.04
RP11-7F17.8	1.05	2.28	0.46	0.01
SPRY4-IT1	2.40	5.21	0.46	0.04
RP11-803D5.1	1.15	2.51	0.46	0.01
RP11-815I9.4	1.54	3.40	0.45	0.02
AC009120.6	9.51	21.09	0.45	0.02
RP11-88I18.2	2.07	4.60	0.45	0.01
RP11-334J6.7	8.26	18.38	0.45	0.00
RP5-875O13.1	1.05	2.35	0.45	0.03
RP11-73M18.7	2.56	5.72	0.45	0.04
AC092669.3	2.24	5.05	0.44	0.01
CTD-2574D22.3	0.98	2.21	0.44	0.02
RP11-706O15.1	4.15	9.39	0.44	0.02
UGDH-AS1	1.95	4.46	0.44	0.00
CTD-2291D10.4	2.88	6.57	0.44	0.04
BCYRN1	0.97	2.24	0.43	0.00
RP11-295D4.1	1.06	2.45	0.43	0.01
CASC2	2.53	5.86	0.43	0.03
CTC-250I14.6	32.48	75.34	0.43	0.00
PRKAR2A-AS1	3.03	7.04	0.43	0.01
RP11-382B18.1	0.97	2.26	0.43	0.01
LINC00667	3.05	7.09	0.43	0.03

RP1-117B12.4	22.93	53.48	0.43	0.00
RP11-500C11.3	22.35	52.37	0.43	0.00
RP11-274B21.14	1.73	4.10	0.42	0.01
AC092171.4	1.68	3.97	0.42	0.00
AC005786.7	8.84	21.11	0.42	0.01
RP11-843B15.4	4.09	9.81	0.42	0.02
CTC-338M12.4	2.83	6.81	0.42	0.02
AC006547.15	2.31	5.58	0.41	0.03
HCG18	1.16	2.88	0.40	0.00
RP11-306O13.1	1.19	2.94	0.40	0.01
CTD-2231E14.2	3.33	8.26	0.40	0.00
CTB-31O20.3	4.22	10.48	0.40	0.02
CTB-31O20.9	1.72	4.27	0.40	0.04
AC005682.5	1.46	3.64	0.40	0.05
RP11-635L1.2	0.96	2.42	0.40	0.05
RP11-138A9.1	1.04	2.64	0.40	0.01
RP11-196G11.3	1.43	3.63	0.39	0.00
AC007743.1	0.93	2.37	0.39	0.00
RP11-445F12.2	2.36	6.03	0.39	0.02
LLNLR-284B4.1	15.25	39.04	0.39	0.00
PSMA3-AS1	1.22	3.13	0.39	0.03
RP11-263K19.6	1.62	4.15	0.39	0.03
RP11-706O15.3	1.18	3.05	0.39	0.01

RP11-103H7.5	1.73	4.52	0.38	0.04
LINC01551	0.88	2.30	0.38	0.01
RP11-580I16.2	3.54	9.34	0.38	0.02
EAF1-AS1	0.88	2.33	0.38	0.03
NOP14-AS1	2.67	7.35	0.36	0.00
XXbac-BPG181M17.6	4.16	11.44	0.36	0.00
RP11-259O2.3	7.87	21.93	0.36	0.00
RP4-742J24.2	0.85	2.42	0.35	0.00
RP11-801F7.1	3.24	9.28	0.35	0.00
RP11-627G18.1	1.54	4.52	0.34	0.01
RP11-73M18.6	0.94	2.79	0.34	0.05
CTD-2510F5.4	6.56	19.46	0.34	0.01
WNT5A-AS1	1.38	4.15	0.33	0.03
CTC-458I2.2	0.85	2.56	0.33	0.03
RP11-256P1.1	10.40	31.75	0.33	0.00
RP11-442H21.2	6.52	20.00	0.33	0.00
AC017002.2	2.31	7.12	0.32	0.00
U91328.21	2.24	6.96	0.32	0.04
AC079305.10	2.26	7.10	0.32	0.01
CTD-3193O13.13	1.80	5.76	0.31	0.02
MALAT1	163.48	524.77	0.31	0.00
RP11-385F5.5	0.86	2.76	0.31	0.04
NAV2-AS1	1.79	5.95	0.30	0.00

RP11-618G20.1	3.00	9.98	0.30	0.00
RP11-20G6.1	1.29	4.43	0.29	0.02
RP11-530C5.1	19.62	67.90	0.29	0.00
RP11-732A21.3	1.17	4.09	0.28	0.00
MBNL1-AS1	0.79	2.79	0.28	0.01
LINC00969	0.87	3.21	0.27	0.00
PKD1P6	0.92	3.40	0.27	0.02
CTC-436P18.1	2.06	7.61	0.27	0.00
RP11-109P14.9	0.86	3.24	0.26	0.00
UXT-AS1	1.93	7.53	0.26	0.00
RP11-334E6.10	1.82	7.13	0.26	0.00
RP11-489E7.4	2.15	8.46	0.25	0.00
SNHG17	2.10	8.43	0.25	0.00
RP11-455O6.8	1.14	4.67	0.25	0.00
LINC01184	0.98	4.05	0.24	0.01
RP11-45M22.2	0.71	3.01	0.24	0.00
RP11-96L14.7	1.41	6.02	0.23	0.00
RP11-452K12.7	2.11	9.19	0.23	0.00
CH17-189H20.1	0.92	4.97	0.19	0.00
AC005262.2	4.75	26.61	0.18	0.00
RP11-365N19.2	0.56	3.41	0.16	0.00
SNHG1	0.74	4.59	0.16	0.00
RP11-867G23.4	2.65	18.27	0.15	0.00

AL109763.2	0.39	2.83	0.14	0.00
CTBP1-AS2	0.30	2.40	0.13	0.00
SATB2-AS1	0.21	2.43	0.08	0.03
CTD-2135D7.2	0.00	3.24	0.00	0.00
CTD-2349P21.7	0.00	43.81	0.00	0.01
DANCR	0.00	12.36	0.00	0.00
HOXB-AS3	0.00	2.94	0.00	0.02
LINC00963	0.00	7.08	0.00	0.00
LINC01535	0.00	2.95	0.00	0.00
RP11-290M5.4	0.00	6.15	0.00	0.04
RP11-373D23.2	0.00	2.93	0.00	0.04
TUG1	0.00	2.27	0.00	0.00
ZEB1-AS1	0.00	3.39	0.00	0.00

Table S2. NONO binding motifs in glycolytic pre-mRNA

Motif ID	Gene	Start	End	p value	Matched sequence
AGGGA	GCK	49	53	0.000977	aggga
AGGGA	ALDOA	55	59	0.000977	aggga
AGGGA	ALDOA	64	68	0.000977	aggga
AGGGA	SLC2A1	67	71	0.000977	AGGGA
AGGGA	ALDOA	71	75	0.000977	aggga
AGGGA	ALDOA	92	96	0.000977	aggga
AGGGA	ALDOA	104	108	0.000977	aggga
AGGGA	LDHA	106	110	0.000977	aggga
AGGGA	GCK	124	128	0.000977	aggga
AGGGA	PGK1	138	142	0.000977	AGGGA
AGGGA	LDHA	163	167	0.000977	aggga
AGGGA	GCK	275	279	0.000977	aggga
AGGGA	GCK	372	376	0.000977	aggga
AGGGA	PGK1	517	521	0.000977	AGGGA
AGGGA	HK2	525	529	0.000977	AGGGA
AGGGA	LDHA	599	603	0.000977	aggga
AGGGA	HK2	710	714	0.000977	AGGGA
AGGGA	LDHA	759	763	0.000977	aggga
AGGGA	LDHA	786	790	0.000977	aggga
AGGGA	GCK	811	815	0.000977	AGGGA

AGGGA	GCK	920	924	0.000977	AGGGA
AGGGA	SLC2A1	1086	1090	0.000977	aggga
AGGGA	PFKL	1149	1153	0.000977	AGGGA
AGGGA	ALDOA	1251	1255	0.000977	aggga
AGGGA	ALDOA	1309	1313	0.000977	aggga
AGGGA	PGK1	1375	1379	0.000977	AGGGA
AGGGA	GCK	1447	1451	0.000977	AGGGA
AGGGA	GCK	1599	1603	0.000977	AGGGA
AGGGA	PFKL	1662	1666	0.000977	AGGGA
AGGGA	ALDOA	1684	1688	0.000977	aggga
AGGGA	ALDOA	1697	1701	0.000977	aggga
AGGGA	SLC2A1	1771	1775	0.000977	aggga
AGGGA	PGK1	1830	1834	0.000977	AGGGA
AGGGA	SLC2A1	1834	1838	0.000977	aggga
AGGGA	ALDOA	1906	1910	0.000977	aggga
AGGGA	SLC2A1	1955	1959	0.000977	aggga
AGGGA	GCK	2058	2062	0.000977	AGGGA
AGGGA	ALDOA	2058	2062	0.000977	aggga
AGGGA	SLC2A1	2089	2093	0.000977	aggga
AGGGA	PFKL	2239	2243	0.000977	AGGGA
AGGGA	PFKL	2261	2265	0.000977	AGGGA
AGGGA	HK2	2307	2311	0.000977	aggga
AGGGA	HK2	2330	2334	0.000977	aggga

AGGGA	PGK1	2405	2409	0.000977	AGGGA
AGGGA	ALDOA	2787	2791	0.000977	aggga
AGGGA	ALDOA	2861	2865	0.000977	aggga
AGGGA	GCK	2971	2975	0.000977	AGGGA
AGGGA	HK2	3102	3106	0.000977	aggga
AGGGA	ALDOA	3175	3179	0.000977	AGGGA
AGGGA	LDHA	3338	3342	0.000977	AGGGA
AGGGA	ALDOA	3458	3462	0.000977	AGGGA
AGGGA	GCK	3541	3545	0.000977	AGGGA
AGGGA	HK2	3560	3564	0.000977	aggga
AGGGA	LDHA	3573	3577	0.000977	AGGGA
AGGGA	ALDOA	3600	3604	0.000977	AGGGA
AGGGA	LDHA	3774	3778	0.000977	AGGGA
AGGGA	SLC2A1	3906	3910	0.000977	aggga
AGGGA	SLC2A1	4023	4027	0.000977	aggga
AGGGA	ALDOA	4155	4159	0.000977	AGGGA
AGGGA	SLC2A1	4265	4269	0.000977	aggga
AGGGA	ALDOA	4399	4403	0.000977	AGGGA
AGGGA	PFKL	4437	4441	0.000977	AGGGA
AGGGA	PFKL	4447	4451	0.000977	AGGGA
AGGGA	PFKL	4451	4455	0.000977	AGGGA
AGGGA	GCK	4572	4576	0.000977	AGGGA
AGGGA	GCK	4659	4663	0.000977	AGGGA

AGGGA	PFKL	4837	4841	0.000977	AGGGA
AGGGA	ALDOA	4865	4869	0.000977	AGGGA
AGGGA	LDHA	4918	4922	0.000977	AGGGA
AGGGA	GCK	4944	4948	0.000977	AGGGA
AGGGA	SLC2A1	5042	5046	0.000977	aggga
AGGGA	PGK1	5131	5135	0.000977	AGGGA
AGGGA	PFKL	5329	5333	0.000977	AGGGA
AGGGA	PFKL	5459	5463	0.000977	AGGGA
AGGGA	PFKL	5489	5493	0.000977	AGGGA
AGGGA	HK2	5534	5538	0.000977	aggga
AGGGA	ALDOA	5555	5559	0.000977	AGGGA
AGGGA	HK2	5695	5699	0.000977	aggga
AGGGA	HK2	5797	5801	0.000977	aggga
AGGGA	HK2	5809	5813	0.000977	aggga
AGGGA	GCK	5916	5920	0.000977	AGGGA
AGGGA	GCK	6152	6156	0.000977	AGGGA
AGGGA	LDHA	6335	6339	0.000977	AGGGA
AGGGA	GCK	6448	6452	0.000977	AGGGA
AGGGA	GCK	6655	6659	0.000977	AGGGA
AGGGA	GCK	6682	6686	0.000977	AGGGA
AGGGA	PFKL	6731	6735	0.000977	AGGGA
AGGGA	PFKL	6953	6957	0.000977	AGGGA
AGGGA	PGK1	6963	6967	0.000977	AGGGA

AGGGA	PFKL	7093	7097	0.000977	AGGGA
AGGGA	PGK1	7244	7248	0.000977	AGGGA
AGGGA	LDHA	7301	7305	0.000977	AGGGA
AGGGA	LDHA	7363	7367	0.000977	AGGGA
AGGGA	PFKL	7574	7578	0.000977	AGGGA
AGGGA	PGK1	7661	7665	0.000977	AGGGA
AGGGA	GCK	7692	7696	0.000977	AGGGA
AGGGA	SLC2A1	7737	7741	0.000977	aggga
AGGGA	PFKL	8016	8020	0.000977	AGGGA
AGGGA	PFKL	8210	8214	0.000977	AGGGA
AGGGA	PGK1	8216	8220	0.000977	AGGGA
AGGGA	LDHA	8234	8238	0.000977	AGGGA
AGGGA	PFKL	8322	8326	0.000977	AGGGA
AGGGA	SLC2A1	8445	8449	0.000977	aggga
AGGGA	PFKL	8462	8466	0.000977	AGGGA
AGGGA	PGK1	8731	8735	0.000977	AGGGA
AGGGA	PFKL	8934	8938	0.000977	AGGGA
AGGGA	SLC2A1	8950	8954	0.000977	aggga
AGGGA	PFKL	9003	9007	0.000977	AGGGA
AGGGA	HK2	9029	9033	0.000977	aggga
AGGGA	PFKL	9065	9069	0.000977	AGGGA
AGGGA	SLC2A1	9097	9101	0.000977	aggga
AGGGA	PFKL	9143	9147	0.000977	AGGGA

AGGGA	PFKL	9187	9191	0.000977	AGGGA
AGGGA	LDHA	9193	9197	0.000977	AGGGA
AGGGA	HK2	9283	9287	0.000977	aggga
AGGGA	PFKL	9289	9293	0.000977	AGGGA
AGGGA	GCK	9480	9484	0.000977	AGGGA
AGGGA	PGK1	9767	9771	0.000977	AGGGA
AGGGA	PFKL	9814	9818	0.000977	AGGGA
AGGGA	HK2	9823	9827	0.000977	aggga
AGGGA	LDHA	9882	9886	0.000977	AGGGA
AGGGA	PGK1	9883	9887	0.000977	AGGGA
AGGGA	PGK1	9888	9892	0.000977	AGGGA
AGGGA	PFKL	9963	9967	0.000977	AGGGA
AGGGA	HK2	10017	10021	0.000977	aggga
AGGGA	PFKL	10067	10071	0.000977	AGGGA
AGGGA	GCK	10164	10168	0.000977	AGGGA
AGGGA	SLC2A1	10292	10296	0.000977	aggga
AGGGA	PFKL	10401	10405	0.000977	AGGGA
AGGGA	HK2	10507	10511	0.000977	aggga
AGGGA	PFKL	10685	10689	0.000977	AGGGA
AGGGA	SLC2A1	10703	10707	0.000977	aggga
AGGGA	SLC2A1	10745	10749	0.000977	aggga
AGGGA	PFKL	10973	10977	0.000977	AGGGA
AGGGA	HK2	11169	11173	0.000977	aggga

AGGGA	PFKL	11504	11508	0.000977	AGGGA
AGGGA	HK2	11510	11514	0.000977	aggga
AGGGA	SLC2A1	11545	11549	0.000977	aggga
AGGGA	HK2	11583	11587	0.000977	aggga
AGGGA	GCK	11664	11668	0.000977	AGGGA
AGGGA	PGK1	11727	11731	0.000977	AGGGA
AGGGA	SLC2A1	11836	11840	0.000977	aggga
AGGGA	HK2	11852	11856	0.000977	aggga
AGGGA	PFKL	11887	11891	0.000977	AGGGA
AGGGA	PGK1	11961	11965	0.000977	AGGGA
AGGGA	HK2	12062	12066	0.000977	aggga
AGGGA	PFKL	12098	12102	0.000977	AGGGA
AGGGA	GCK	12116	12120	0.000977	AGGGA
AGGGA	PGK1	12179	12183	0.000977	AGGGA
AGGGA	GCK	12283	12287	0.000977	AGGGA
AGGGA	PFKL	12322	12326	0.000977	AGGGA
AGGGA	PFKL	12329	12333	0.000977	AGGGA
AGGGA	PFKL	12449	12453	0.000977	AGGGA
AGGGA	SLC2A1	12594	12598	0.000977	aggga
AGGGA	GCK	12629	12633	0.000977	AGGGA
AGGGA	SLC2A1	12735	12739	0.000977	aggga
AGGGA	PGK1	12739	12743	0.000977	AGGGA
AGGGA	PGK1	12854	12858	0.000977	AGGGA

AGGGA	PGK1	12864	12868	0.000977	AGGGA
AGGGA	GCK	13220	13224	0.000977	AGGGA
AGGGA	HK2	13388	13392	0.000977	aggga
AGGGA	LDHA	13399	13403	0.000977	aggga
AGGGA	PFKL	13522	13526	0.000977	AGGGA
AGGGA	LDHA	13587	13591	0.000977	aggga
AGGGA	PGK1	13678	13682	0.000977	AGGGA
AGGGA	PGK1	13764	13768	0.000977	AGGGA
AGGGA	PFKL	13800	13804	0.000977	AGGGA
AGGGA	SLC2A1	13942	13946	0.000977	aggga
AGGGA	SLC2A1	14020	14024	0.000977	aggga
AGGGA	GCK	14021	14025	0.000977	AGGGA
AGGGA	SLC2A1	14044	14048	0.000977	aggga
AGGGA	PFKL	14309	14313	0.000977	AGGGA
AGGGA	PGK1	14401	14405	0.000977	AGGGA
AGGGA	PGK1	14414	14418	0.000977	AGGGA
AGGGA	PGK1	14469	14473	0.000977	AGGGA
AGGGA	PGK1	14477	14481	0.000977	AGGGA
AGGGA	PFKL	14534	14538	0.000977	AGGGA
AGGGA	PGK1	14577	14581	0.000977	AGGGA
AGGGA	PGK1	14765	14769	0.000977	AGGGA
AGGGA	GCK	14795	14799	0.000977	AGGGA
AGGGA	GCK	14803	14807	0.000977	AGGGA

AGGGA	GCK	14827	14831	0.000977	AGGGA
AGGGA	GCK	14837	14841	0.000977	AGGGA
AGGGA	GCK	14847	14851	0.000977	AGGGA
AGGGA	GCK	14853	14857	0.000977	AGGGA
AGGGA	PGK1	15024	15028	0.000977	AGGGA
AGGGA	PFKL	15111	15115	0.000977	AGGGA
AGGGA	GCK	15173	15177	0.000977	AGGGA
AGGGA	HK2	15225	15229	0.000977	aggga
AGGGA	SLC2A1	15247	15251	0.000977	aggga
AGGGA	PFKL	15326	15330	0.000977	AGGGA
AGGGA	PGK1	15374	15378	0.000977	AGGGA
AGGGA	HK2	15926	15930	0.000977	aggga

Table S3. Small interfering RNA (siRNA)

Target gene	Strand	Sequence(5’->3’)
NEAT1	Sense	CUGGUAUGUUGCUCUGUAUGGUAdAdG
	Antisense	CUUACCAUACAGAGCAACAUACCAGUA
NEAT1_2	Sense	GGGUAAAUCUCAAUCUUAAdTdT
	Antisense	UUAAGAUUGAGAUUUACCCdAdG
NONO-1	Sense	GCAUUCCUGAAGUCUCUAAdTdT
	Antisense	UUAGAGACUUCAGGAAUGCdTdT
NONO-2	Sense	GCCAGAAUUCUACCCUGGAAAdTdT
	Antisense	UUUCCAGGGUAGAAUUCUGGCdTdT
siNC	Sense	UUCUCCGAACGUGUCACGUTT
	Antisense	ACGUGACACGUUCGGAGAATT

Table S4. DNA primers for quantification of gene expression

Target	Strand	Sequence(5'->3')
NEAT1	Forward	CAATTACTGTCGTTGGGATTTAGAGTG
	Reverse	TTCTTACCATACAGAGCAACATACCAG
NEAT1-2	Forward	CAGTTAGTTTATCAGTTCTCCCATCCA
	Reverse	GTTGTTGTCGTCACCTTTCAACTCT
NONO	Forward	GGCAGGCGAAGTCTTCATTCA
	Reverse	TGGCAATCTCCGCTAGGGT
ACTIN	Forward	TCCCTGGAGAAGAGCTACGA
	Reverse	AGCACTGTGTTGGCGTACAG
GAPDH	Forward	CGAGATCCCTCCAAAATCAA
	Reverse	TTCACACCCATGACGAACAT
GLUT1	Forward	ACCATTGGCTCCGGTATCG
	Reverse	GCTCGCTCCACCACAAACA
Unspliced GLUT1	Forward	ACCATTGGCTCCGGTATCG
	Reverse	GCTGTGGGCAGAGACAGTGT
HK2	Forward	ACCCGGGAAAGCAACTGTTTG
	Reverse	TCACCAGGATAAGCCTCACCAG
Unspliced HK2	Forward	GAAGTTGCACGTGTGCGCAT
	Reverse	TCACCAGGATAAGCCTCACCAG
PGK1	Forward	GACCTAATGTCCAAAGCTGAGAA
	Reverse	CAGCAGGTATGCCAGAAGCC
Unspliced PGK1	Forward	GACCTAATGTCCAAAGCTGAGAA

	Reverse	CTACCAACCCACTCAAGTGA
LDHA	Forward	ATGGCAACTCTAAAGGATCAGC
	Reverse	CCAACCCCAACAACTGTAATCT
Unspliced LDHA	Forward	ATGGCAACTCTAAAGGATCAGC
	Reverse	CAGTGTGGTAGACTCTCACTTAC

Table S5. DNA primers for constructing NEAT1 plasmids

Expression Construct	Strand	Sequence(5'->3')
pCDNA3.1-NEAT1_2	Forward	ATTGGTACCGGAGTTAGCGACAGGGAGGG<br>ATGCGCGCCT
	Reverse	GACTCTAGACTAATGAGTTTAGAACTCAAA<br>CTTTATTTGTG
penter1A-shNEAT1_2	Forward	TCAGTCGACTGGATCCCCGGACAGCATACT<br>GGCCAGAAA CTCGAG<br>TTTCTGGCCAGTATGCTGT TTTTTG<br>GATATCTAGACCCAGCT
	Reverse	AGCTGGGTCTAGATATCCAAAAAACAGCAT<br>ACTGGCCAGAAACTCGAGTTTCTGGCCAGT<br>ATGCTGTCCGGGGATCCAGTCGACTGA

Table S6. Primers for ChIP-PCR

Target	Strand	Sequence(5’->3’)
mNEAT1	Forward	CTGGTTCAAGCACGCAGAAT
	Reverse	TGCTGGTCACATGAGAGAG
hGAPDH	Forward	CCACAGTCCAGTCCTGGGAACC
	Reverse	GAGCTACGTGCGCCCGTAAAA
hNEAT1-1	Forward	TGGAGGAGTCAGGAGGAATA
	Reverse	GCATGGACAAGTTGAAGAT
hNEAT1-2	Forward	GACTCCTCCATTGGTTTCATAAG
	Reverse	CAGTCTCTTCCTGACTGTAT
hNEAT1-3	Forward	GTCAGAATCAAGAGATGTTAG
	Reverse	CAACTCTAACCGGCCTCTTC

Supplementary Material and Methods

Cell growth assay

The growth of liver cancer cells was determined by optimized sulforhodamine B (SRB) assay as described [1]. Briefly, cells with different treatment were seeded in triplicate in 96-well plates at an initial density of 1×10^3 cells/well. Then, the cells were then fixed with 10% (wt/vol) trichloroacetic acid for 1 h and stained with SRB solution (0.057% w/v in 1% acetic acid) for 30 min. Following the SRB staining, 1% (vol/vol) acetic acid was used to remove the excess dye. Finally, the protein-bound dye was dissolved in 10 mM Tris solution for OD determination at 492 nm using a microplate reader. The relative growth was measured daily for 5 consecutive days.

Immunological reagents, immunoprecipitation and immunoblot

The following immunological reagents were used in this study. Antibodies against GAPDH (#2118), ACTB(#4970), p-S6 (S235/236), S6 (#2217), p-S6K (T398), S6K, p-AKT(T308) (#13038), AKT (#4691), mTOR (#2983), GLUT1 (#73015), HK2 (#2867), PGK1(#68540), LDHA (#3582)), β-Catenin (#8480), Arginase-1 (#93668), Stat3 (#12640) and p-Stat3(S727) (#34911) were purchased from Cell Signaling Technology (USA). Ki-67 (#Ab16667) antibodies and CK-19 (#Ab133496) were purchased from Abcam Inc. (USA). Antibodies against NONO (N8789), EFTUD2 (#HPA022021) were purchased from Sigma (USA). Antibodies against NONO (#66361-1-Ig), SFPQ (#15585-1-AP), PRPF6 (#23929-1-AP), PRPF8 (#11171-1-AP) and BRR2 (#23875-1-AP) were purchased from Proteintek (USA). Antibody against Glypican 3 (#LS-B11249) was purchased from LifeSpan Biosciences (USA).

SNU423 cells were harvested 2 h after replacement of fresh medium, and lysed in immunoprecipitation buffer (25 mM Tris-HCl pH 7.4, 150 mM NaCl, 1 mM EDTA, 1% NP-40 and 5% glycerol) with 2.5 mg/ml DSP and 1× protease inhibitor cocktail (Sigma, USA). After incubation for 30 min at 4°C with gentle agitation, 1M Tris-HCl PH7.4 was used to end crosslinking reaction. Then the supernatants were collected by centrifugation at 12,000 × g for 15 min at 4°C. Cell lysates were incubated with IP antibody at 4°C

overnight and then with protein A/G-agarose beads (Thermo Fisher Scientific, USA) for another 2 h at 4°C. The pellets were washed with immunoprecipitation wash buffer (25 mM Tris-HCl (pH 7.4), 150 mM NaCl, 1 mM EDTA, 1% Triton and 5% glycerol) for six times, boiled in protein sample buffer and analyzed by immunoblot.

Duolink proximal ligation assay (PLA)

Duolink assay was carried out based on the manufacturer's instruction. SNU423 cells with different treatments were cultured on Chamber-slides (#154526, Thermo Fisher Scientific), and fixed using 4% paraformaldehyde after cultured for 24 h. Cells were blocked with blocking solution (Sigma, USA) and then incubated with primary antibodies at 4°C overnight. Antibodies against NONO (mouse) and EFTUD2 (rabbit) that from different species were utilized. A single antibody was used as a negative control. Duolink® In Situ PLA® Probe AntiMouse MINUS with Duolink® In SituDetection Reagents Red and Duolink® In Situ PLA® Probe Anti-Rabbit PLUS and (DUO92008, DUO92002, DUO02004, Sigma-Aldrich, USA) were used for amplification and detection of bound PLA probes. All Chamber-slides were mounted using Mounting Media with 4',6-diamidino-2-phenylindole (DAPI). Images were acquired using an Olympus fluorescence microscope. The densities of NONO within NEAT1 interacting signals were calculated from fluorescence intensities.

RNA preparation and quantitative reverse transcription PCR (qRT-PCR)

Total RNA was isolated using Trizol (Thermo Fisher Scientific, USA based on manufacturer's instructions. Following digestion with RNase-free DNase I (Thermo Fisher Scientific, USA), 500 ng RNA was subjected to reverse transcription to synthesize cDNA using random-hexamer primers (Takara) and M-MLV Reverse Transcriptase (Takara). Complementary DNAs diluted 1:100 was analyzed using the SYBR Green PCR Master Mix (RNase-free DNase I (Thermo Fisher Scientific, USA). To specifically quantify NEAT1_1, cDNA was reverse-transcribed using oligo (dT)15 primers. The details of primer sequence are listed in in the Table S3. Data were collected and analyzed using a QuantStudio 12K Flex Real-Time PCR System

(Thermo Fisher Scientific, USA). The housekeeping gene beta-Actin was used for normalization. Unspliced RNA transcripts will be detected using PCR primers flanking an exon-intron junction. Intron retention = (Expression of unspliced RNA transcripts) / (Expression of intron-excluded region).

RNA-seq and analysis of differentially expressed lncRNAs

SNU423 cells were treated with 100 nM rapamycin (Selleck Chemicals, USA) or the vehicle DMSO for 2 h. After washing with ice-cold PBS, RNA was prepared using the Trizol protocol and treated with RNase-free DNase I (Thermo Fisher Scientific, USA). LncRNA library construction, RNA quality assessment, and sequencing (Hiseq4000 Sequencing platform) were performed by Novogene. Differentially expressed lncRNAs after rapamycin treatment were identified using the following procedure: raw data were first preprocessed to obtain clean data by removing sequencing adapter and low quality reads using perl scripts. Sequencing reads were aligned to the human reference genome through HISAT2 (version 2.0.4) [2]. Then Stringtie [3] was utilized for assembly of lncRNA transcripts. After basic lncRNA filtering through Cufflinks and coding potential filtering (using CPC, PfamScan, CNCI, PhyloCSF), provisional lncRNAs were determined. Cufflmerge and Cuffdiff (version 2.0.4) were then applied to calculate the RPKM (reads per kilobase per million) values and determine differentially expressed lncRNAs between control and rapamycin treated groups. We only considered non-coding RNAs that expressed at relative high levels (RPKM > 2) and showed significant changes (|log2 fold change (FC)| > 1, adjusted P < 0.05) between control and treatment cells. 202 differentially expressed lncRNAs were identified.

Raw RNA sequencing data generated from this study has been deposited in Sequence Read Archive (SRA) of the NCBI under the access number SRP314313 that can be accessed through the following link: https://dataview.ncbi.nlm.nih.gov/object/PRJNA721096?reviewer=mm3edm3d3g2bmi8l5mg5goq5l0.

Chromatin immunoprecipitation (ChIP) assay

Cells (3 × 107) were suspended in 3.7% formaldehyde at room temperature for 10 min and then blocked by addition of glycine to a final concentration of 0.25 M for 5 min on ice. After centrifugation at 1,000 r.p.m.

for 5 min, cell pellets were resuspended in 500 µl lysis buffer (50 mM Tris pH 8.0, 5 mM EDTA, 150 mM NaCl, 1% Triton X-100, 0.1% sodium deoxycholate, proteinase inhibitor cocktail). Chromatin shearing was performed by sonication with Diagenode's Bioruptor® (Diagenode, USA) to generate 300–600 bp DNA fragments, which were collected in the supernatant after centrifugation at 12,000 g for 15 min at 4°C. The supernatant was incubated with anti-STAT3 antibody (CST, USA) at 4°C overnight and then with protein A/G-agarose beads (Thermo Fisher Scientific, USA) for another 3 h at 4°C. Following the incubation, the beads were washed with 600 µl SDS lysis buffer (50 mM Tris-HCl at pH 8.0, 10 mM EDTA, 0.5 M NaCl, 1% SDS), following with 600 µl TE Buffer (10 mM Tris pH 8.0, 1 mM EDTA) for twice at 4°C. Finally, the protein-DNA complex was eluted by adding 150 µl freshly prepared elution buffer (50 mM Tris pH 8, 10 mM EDTA, 1% SDS). Reverse crosslinking was performed by adding NaCl and incubating at 65 °C for 4 h, followed by a supplement of EDTA and proteinase K at 55 °C for 2 h. DNA was purified for PCR analysis. Primers are listed in the Table S3.

Splicing reporter assay

4×10^4 SNU423 cells were plated in a 12-well plate. After culturing overnight, cells were co-transfected with NEAT1_2-specific siRNA and the pTN24 splicing reporter plasmid, which expresses a functional luciferase upon removal of a translation termination signal sequence by splicing, and expresses constitutive β-galactosidase reporter for normalization. 48 h after transfection and rapamycin treatment, cells were harvested for detection of reporter expression using the Dual Light Reporter System (Applied Biosystems) as previously described [4]. Data was analyzed by calculating the ratio of luciferase to β-galactosidase signals. Statistical analysis was performed with Student’s t-test.

RNAscope assay

RNAscope was performed based on the manufacturer’s instructions (Advanced Cell Diagnostics, Newark, CA, USA) and the RNAscope probe targeting NEAT1-2 was designed and synthesized by Advanced Cell Diagnostics company. After baking, paraffin-embedded liver sections (4-µm thick) were dewaxed in xylene

and 100% ethyl alcohol for 2 min each and treated with PBS for 10 min, followed by treatment with RNAscope® Target Retrieval Reagents and hydrogen peroxide (Advanced Cell Diagnostics, Newark, CA, USA). Probes were then added and incubated for 2 h at 40°C in a humidity control chamber. Signal amplification and detection reagents (Advanced Cell Diagnostics, Newark, CA, USA) were sequentially applied and incubated in AMP 1, AMP 2, AMP 3, AMP 4, AMP 5, and AMP 6 reagents, for 30, 15, 30, 15, 30, 15 min, respectively. Detection of NEAT1_2 expression signals was performed using an RNAscope 2.5 High Definition (HD)-BROWN Detection Reagent (Advanced Cell Diagnostics, Newark, CA, USA). The slides were then counterstained with 50% hematoxylin I (Sigma, USA) for 2 min at room temperature followed by tap water rinse. Finally, slides were then dehydrated with 70%, 80%, 95% and 100% ethyl alcohol gradients for 2 min each, followed by xylene treatment for 5 min. Mounting media and cover slips were then added to each slides for imaging. The images were acquired with an Olympus microscope.

RNA fluorescence in situ hybridization (RNA-FISH) and immunofluorescence staining

NEAT1_2 RNA–FISH was carried out using Stellaris FISH probes (human, SMF-2037-1; mouse, SMF-3010-1) according to the manufacturer's protocol. Briefly, cells were rinsed with ice-cold PBS and then fixed in 4% formaldehyde in PBS (pH 7.4) for 15 min at room temperature. Cells were next immersed in 70% ethanol for at least 1 h at 4°C, followed by permeabilization in PBS containing 0.25% Triton X-100 for 10 min. Hybridization was subsequently performed using Stellaris® FISH Probes (LGC Biosearch Technologies, USA) at 37°C overnight in a moist chamber. Cells were washed with PBS and then counterstained with DAPI. Finally, cells were mounted with Vectashield Anti-Fade mounting medium (Vector Laboratories). For simultaneous immunofluorescence staining of NONO, after RNA-FISH, cells were again fixed for 15 min in 1% formaldehyde and immunofluorescence staining were performed using NONO primary antibody (Sigma, USA). Images were acquired in multi-tracking mode using an Olympus scanning. NEAT1_2 or NEAT1_2-NONO quantification was performed by taking at least 25 cells per group at a 60× magnification and densities of NONO within NEAT1_2 containing speckles were calculated from fluorescence signals.

Gene expression and survival analysis in TCGA HCC dataset

The expression of lncRNA candidates in tumors and normal tissues were analyzed using the HCC dataset (369 primary HCC and 160 normal liver tissues) of GEPIA database. mRNA expression data of NEAT1_2 and NEAT1_1 transcript was analyzed and downloaded from UCSC Xena platform (https://xenabrowser.net/) [5]. The expression data are first log2 (TPM+1) transformed and the differential analysis here is based on the selected datasets ("TCGA tumors vs TCGA normal + GTEx normal"). Further overall survival analysis was performed with high and low NEAT1 expression in 371 primary HCC tissues to evaluate the correlation of NEAT1 expressions with HCC patients overall survival.

Glucose uptake, lactate production and Seahorse assays

SNU423 cells were seeded at 2×10^5 cells/ml into 6-well plates and transfected with specific siRNA against NEAT1_2 with Lipofectamine 3000 (Thermo Fisher Scientific, USA) based on the manufacturer's instructions. Cells were trypsinized and seeded into black-walled clear-bottomed 96-well plates (Costar, USA). Cells were then changed with fresh culture medium with 100 nM rapamycin for 24 h and measured for glucose uptake (#J1341, Promega) and lactate production (#J5021, Promega) according to manufacturer's instructions.

The extracellular acidification rate (ECAR) was determined using a XF24 Extracellular Flux Analyzer (Seahorse Bioscience) based on the manufacturer's instructions. Briefly, cells were plated at 2×10^5 cells per ml in 6-well plates and transfected with specific siRNA against NEAT1 with Lipofectamine 3000 (Thermo Fisher Scientific, USA) according to the manufacturer's instructions. Then cells were trypsinized and seeded into 24-well Seahorse XF24 culture plates with 10% fetal bovine serum RMPI-1640 and 100 nM rapamycin was added to the medium to inhibits mTOR activity. After indicated treatments , cells were incubated in base medium with 2 mM L-glutamine at 37 °C for 1 h and ECAR assay were performed in a CO2-free incubator to ensure accurate measurements of extracellular pH. After every 3 measurements in

8-min intervals, 10 mM glucose, 10 μM oligomycin or 50 mM 2-deoxy-D-glucose (Sigma, USA) was added at the indicated time points into each well. Finally, glycolytic capacity was calculated as the difference between the ECAR following the injection of 1 μm oligomycin and the basal ECAR reading. ECAR values are presented as the mean ± s.d. of experimental triplicates.

LC-MS measurement of [U-^{13}C]-glucose labeled metabolites

For isotope tracing of glycolytic metabolites in HCC cells, 0.5 × 10^6 SNU423 cells were plated and transfected with specific siRNA against NEAT1_2 with Lipofectamine 3000 (Thermo Fisher Scientific, USA) based on the manufacturer's instructions. After treatment with 100 nM rapamycin for 16 h, the medium was changed to glucose-free DMEM containing 10% dialyzed serum and 25 mM [U-^{13}C]-glucose and incubated for 15 min. Metabolites were extracted in ice-cold methanol:acetonitrile:water (40:40:20) with 0.5% formic acid and then neutralized with 15% NH4HCO3. The metabolite extracts were analyzed using a Q Exactive PLUS Hybrid Quadrupole-Orbitrap Mass Spectrometer (Thermo Fisher Scientific) coupled to hydrophilic interaction chromatography (HILIC). The mass spectrometer was under a negative ionization mode. Glycolytic metabolite features were extracted in MAVEN with a 5 ppm-mass accuracy window. The ^{13}C isotope abundance and impurity of labeled substrate were corrected and normalized using AccuCor written in R.

Pathway enrichment analysis of CCLE metabolomic dataset of HCC cells

Metabolomics and NEAT1 mRNA expression datasets of HCC cell lines were obtained from the CCLE portal (https://portals.broadinstitute.org/ccle). 22 metabolites that negatively correlated with NEAT1 expression were uploaded in metaboanalyst (http://www.metaboanalyst.ca/MetaboAnalyst/). Metabolite Set Enrichment Analysis (MSEA) was subsequently carried out using Metaboanalyst (http://www.metaboanalyst.ca/MetaboAnalyst/) and the KEGG (http://www.genome.jp/kegg/pathway.html) pathway database. Metabolic pathways negatively correlated with NEAT1 expression was generated. The overrepresentation analysis (ORA) tool was used to determine

whether a particular pathway was significant. Only metabolite sets with statistically significant ($p < 0.05$) subsystems were shown.

NONO motif analysis

The consensus NONO RNA-binding motif M307_0.6 (AGGGA) from the Database of RNA-binding protein specificities (RBPDB) was utilized for the motif scanning analysis. NONO motif loci analysis was carried out using Find Individual Motif occurrences (FIMO, v5.3.3) to identify statistically overrepresented motifs using default parameters. The motifs identified in the input RNA molecules that correlated with NEAT1 expression in TCGA hepatocellular carcinoma (LIHC) transcriptome dataset are sorted based on the one-sided Fisher's Test p-value ($p < 0.0001$) and details of these candidate regions are listed in the Table S2.

References

1. Vichai V, Kirtikara K. Sulforhodamine B colorimetric assay for cytotoxicity screening. Nat Protoc. 2006; 1: 1112-6.

2. Kim D, Langmead B, Salzberg SL. HISAT: a fast spliced aligner with low memory requirements. Nat Methods. 2015; 12: 357-60.

3. Pertea M, Pertea GM, Antonescu CM, Chang TC, Mendell JT, Salzberg SL. StringTie enables improved reconstruction of a transcriptome from RNA-seq reads. Nat Biotechnol. 2015; 33: 290-5.

4. Nasim MT, Eperon IC. A double-reporter splicing assay for determining splicing efficiency in mammalian cells. Nat Protoc. 2006; 1: 1022-8.

5. Goldman MJ, Craft B, Hastie M, Repecka K, McDade F, Kamath A, et al. Visualizing and interpreting cancer genomics data via the Xena platform. Nat Biotechnol. 2020; 38: 675-8.
